# Supplementary material for: New Fusarin Derivatives from the Marine Algicolous Fungus Penicillium steckii SCSIO41040
Source: Mar Drugs. 2023 Oct 12;21(10):532. doi: 10.3390/md21100532 (PMC10608775; doi:10.3390/md21100532)
Supplement: Supplementary file 1 [file marinedrugs-21-00532-s001.zip › marinedrugs-2663252-supplementary.pdf]

Supporting Information

New Fusarin Derivatives from the Marine Algicolous Fungus *Penicillium steckii* SCSIO41040

Yingying Song [1,2], Jianglian She [1,2], Weihao Chen [1], Jiamin Wang [1,2], Yanhui Tan [3], Xiaoyan Pang [1], Xuefeng Zhou [1,2], Junfeng Wang [1,2,*] and Yonghong Liu [1,2,*]

1 CAS Key Laboratory of Tropical Marine Bio-Resources and Ecology, Guangdong Key Laboratory of Marine Materia Medica, South China Sea Institute of Oceanology, Chinese Academy of Sciences, Guangzhou 510301, China; songyingying19@mails.ucas.ac.cn (Y.S.); shejianglian20@mails.ucas.ac.cn (J.S.); chenweihao17@mails.ucas.ac.cn (W.C.); wangjiamin20@mails.ucas.ac.cn (J.W.); xypang@scsio.ac.cn (X.P.); xfzhou@scsio.ac.cn (X.Z.)

2 University of Chinese Academy of Sciences, Beijing 100049, China

3 State Key Laboratory for Chemistry and Molecular Engineering of Medicinal Resources, School of Chemistry and Pharmaceutical Sciences, Guangxi Normal University, Guilin 541004, China; tyh533@126.com

* Correspondence: wangjunfeng@scsio.ac.cn (J.W.); yonghongliu@scsio.ac.cn (Y.L.)

List of Supporting Information

The ITS gene sequence data of *Penicillium* sp. SCSIO 41040
Figure S1 ^{1}H NMR spectrum of compound **1** in DMSO-d_6
Figure S2 ^{13}C NMR spectrum of compound **1** in DMSO-d_6
Figure S3 DEPT135 spectrum of compound **1** in DMSO-d_6
Figure S4 ^{1}H-^{1}H COSY spectrum of compound **1** in DMSO-d_6
Figure S5 HSQC spectrum of compound **1** in DMSO-d_6
Figure S6 HMBC spectrum of compound **1** in DMSO-d_6
Figure S7 NOESY spectrum of compound **1** in DMSO-d_6
Figure S8 HRESIMS spectrum of compound **1**
Figure S9 UV spectrum of **1**
Figure S10 CD spectrum of **1**

Figure S11 ^{1}H NMR spectrum of compound **2** in DMSO-d_6
Figure S12 ^{13}C NMR spectrum of compound **2** in DMSO-d_6
Figure S13 DEPT135 spectrum of compound **2** in DMSO-d_6
Figure S14 ^{1}H-^{1}H COSY spectrum of compound **2** in DMSO-d_6
Figure S15 HSQC spectrum of compound **2** in DMSO-d_6
Figure S16 HMBC spectrum of compound **2** in DMSO-d_6
Figure S17 NOESY spectrum of compound **2** in DMSO-d_6
Figure S18 HRESIMS spectrum of compound **2**
Figure S19 UV spectrum of **2**
Figure S20 CD spectrum of **2**

Figure S21 ^{1}H NMR spectrum of compound **3** in DMSO-d_6
Figure S22 ^{13}C NMR spectrum of compound **3** in DMSO-d_6
Figure S23 DEPT135 spectrum of compound **3** in DMSO-d_6
Figure S24 ^{1}H-^{1}H COSY spectrum of compound **3** in DMSO-d_6
Figure S25 HSQC spectrum of compound **3** in DMSO-d_6
Figure S26 HMBC spectrum of compound **3** in DMSO-d_6
Figure S27 NOESY spectrum of compound **3** in DMSO-d_6
Figure S28 HRESIMS spectrum of compound **3**
Figure S29 UV spectrum of **3**

Figure S30 ^{1}H NMR spectrum of compound **4** in DMSO-d_6
Figure S31 ^{13}C NMR spectrum of compound **4** in DMSO-d_6
Figure S32 DEPT135 spectrum of compound **4** in DMSO-d_6
Figure S33 ^{1}H-^{1}H COSY spectrum of compound **4** in DMSO-d_6
Figure S34 HSQC spectrum of compound **4** in DMSO-d_6
Figure S35 HMBC spectrum of compound **4** in DMSO-d_6
Figure S36 NOESY spectrum of compound **4** in DMSO-d_6
Figure S37 HRESIMS spectrum of compound **4**

Figure S38 UV spectrum of **4**

Figure S39 ^{1}H NMR spectrum of compound **5** in DMSO-d_6
Figure S40 ^{13}C NMR spectrum of compound **5** in DMSO-d_6
Figure S41 DEPT135 spectrum of compound **5** in DMSO-d_6
Figure S42 ^{1}H-^{1}H COSY spectrum of compound **5** in DMSO-d_6
Figure S43 HSQC spectrum of compound **5** in DMSO-d_6
Figure S44 HMBC spectrum of compound **5** in DMSO-d_6
Figure S45 NOESY spectrum of compound **5** in DMSO-d_6
Figure S46 HRESIMS spectrum of compound **5**
Figure S47 UV spectrum of **5**
Table S1 Energies of **1** and **2** at MMFF94 force field
Table S2 Energies of **1** and **2** at B97-3c level in methanol

The ITS gene sequence data of *Penicillium* sp. SCSIO 41040

GATATGCTTAAGTTCAGCGGGTATCCCTACCTGATCCGAGGTCAACCTGAGAAAAAATA

AAAGGTTGGGGGTCGGCTGGCGCCGGCCGGGCCTACAAGAGCGGGTGACGAAGCCCC

ATACGCTCGAGGACCGGACGCGGTGCCGCCGCTGCCTTTCGGGCCCGTCCCCCCCGGA

GCGGGGGGGACGGGGCCCAACACACAAGCCGTGCTTGAGGGCAGCAATGACGCTCG

GACAGGCATGCCCTCCGGAATACCAGAGGGCGCAATGTGCGTTCAAAGACTCGATGAT

TCACTGAATTCTGCAATTCACATTAGTTATCGCATTTCGCTGCGTTCTTCATCGATGCCG

GAACCAAGAGATCCGTTGTTGAAAGTTTTAACTAATTTAGCTAGTTGTCTCAGACTGCA

ACTTCAGACAGCGTTCAGAGGGGGGCTTCGGCGGGCGCGGGCCCGGGGGCGGATGCC

CCCCGGCGGCCTGGCGGCGGGCCCGCCGAAGCAACACAGGTTCGTGCAACACGGGTG

GGAGGTTGGACCCAGAGGGCCCTCACTCGGTAATGATCCTTCCGCAG

The NMR spectra were uploaded to the Harvard Dataverse (https://doi.org/10.7910/DVN/PXCKSJ).

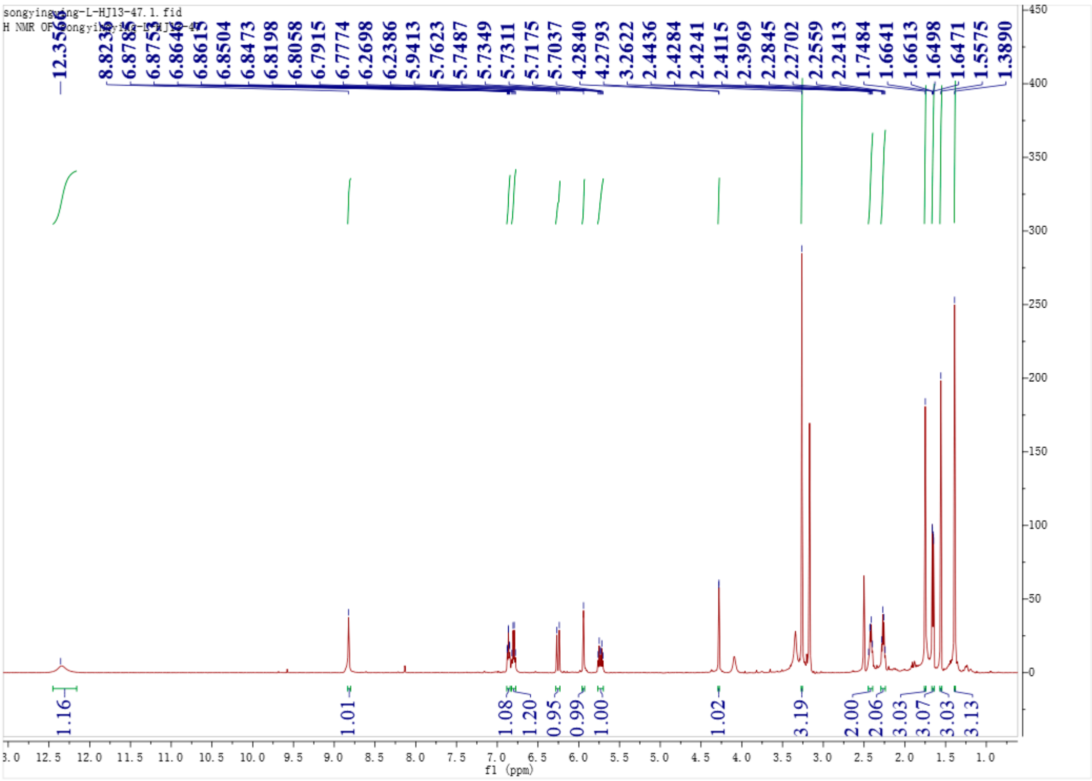

Figure S1 ^{1}H NMR spectrum of compound **1** in DMSO-d_6

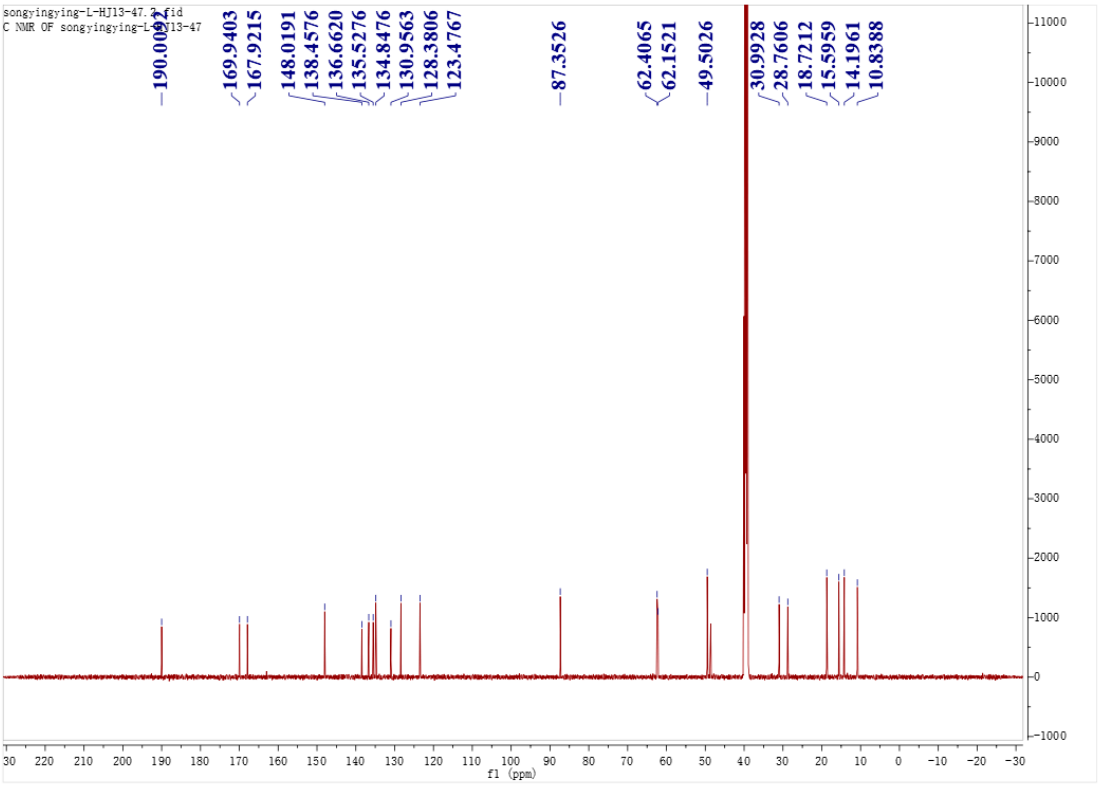

Figure S2 ^{13}C NMR spectrum of compound **1** in DMSO-d_6

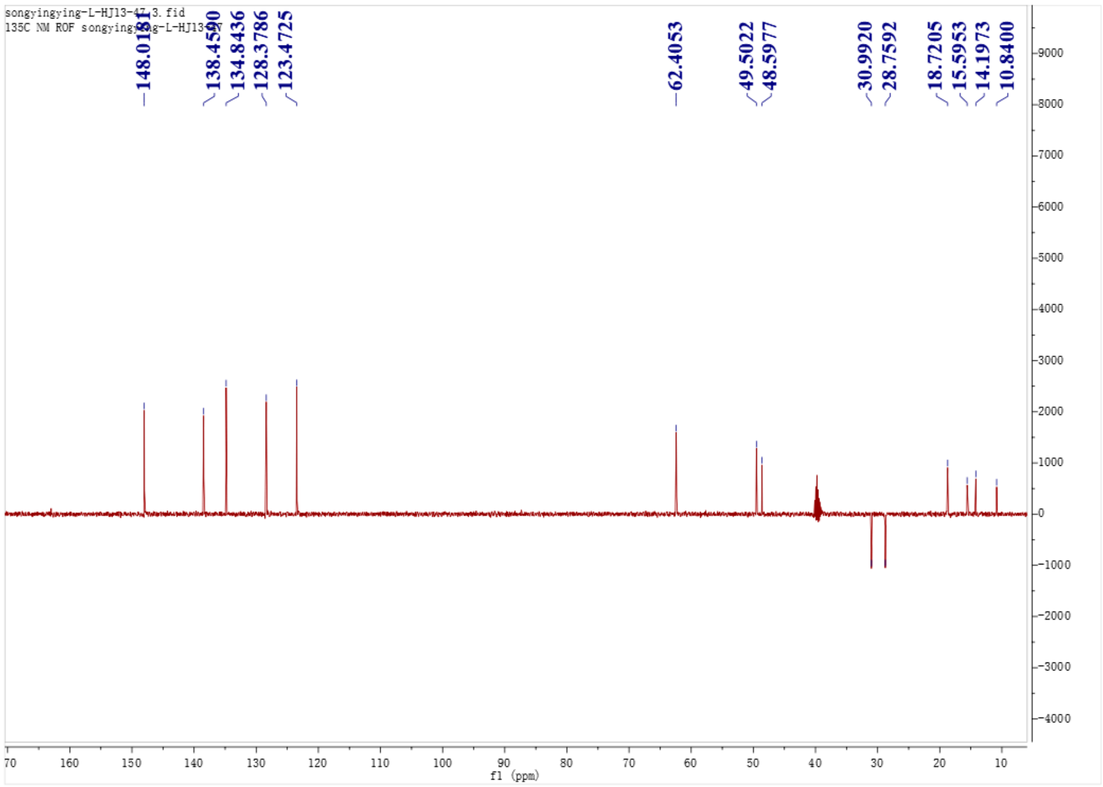

Figure S3 DEPT135 spectrum of compound **1** in DMSO-d_6

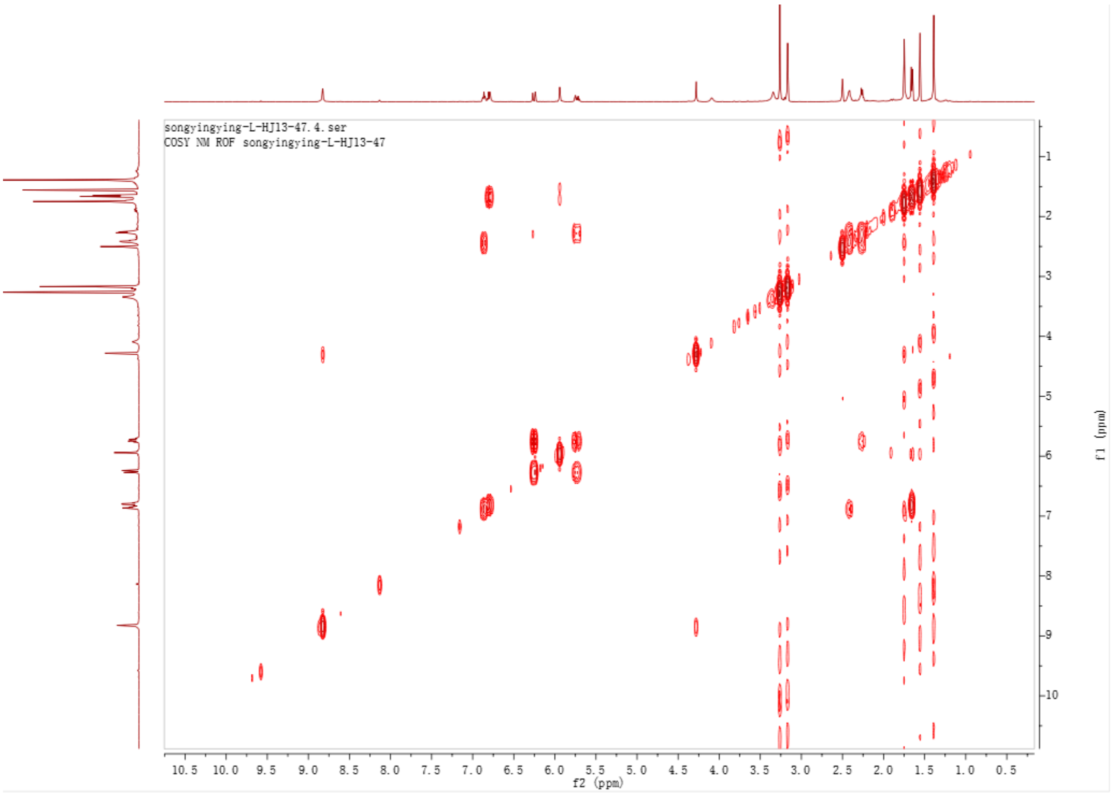

Figure S4 ^{1}H-^{1}H COSY spectrum of compound **1** in DMSO-d_6

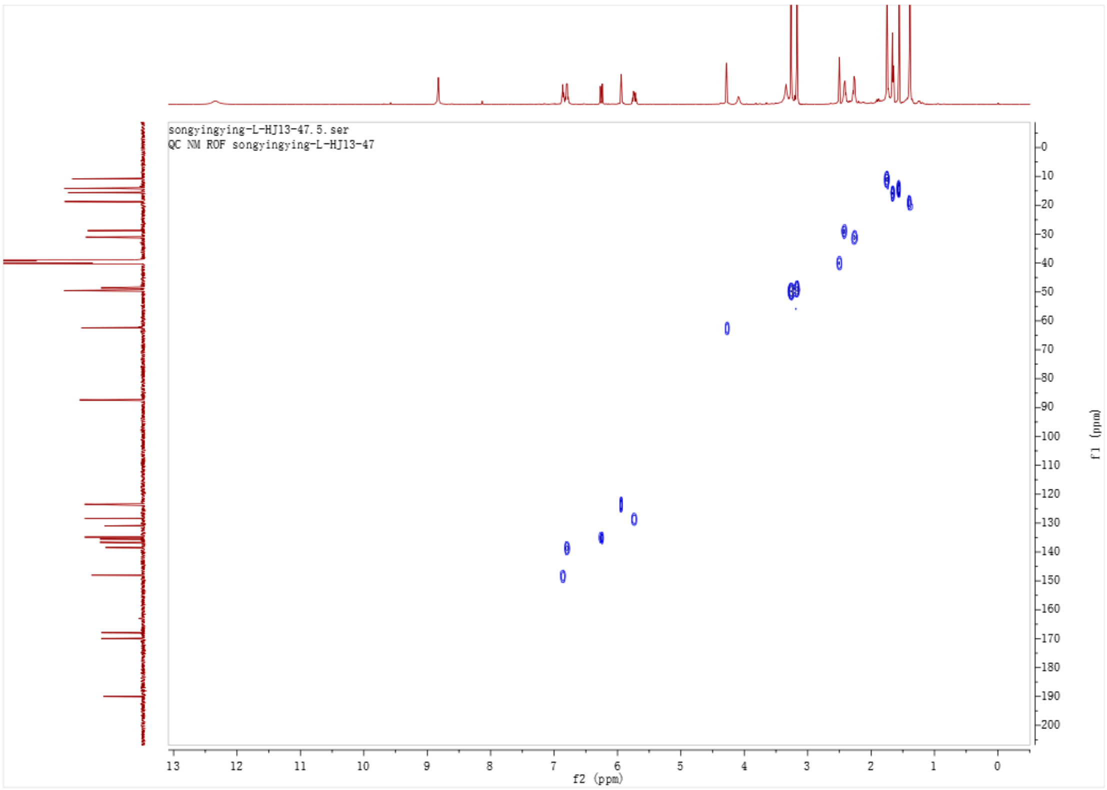

Figure S5 HSQC spectrum of compound **1** in DMSO-d_6

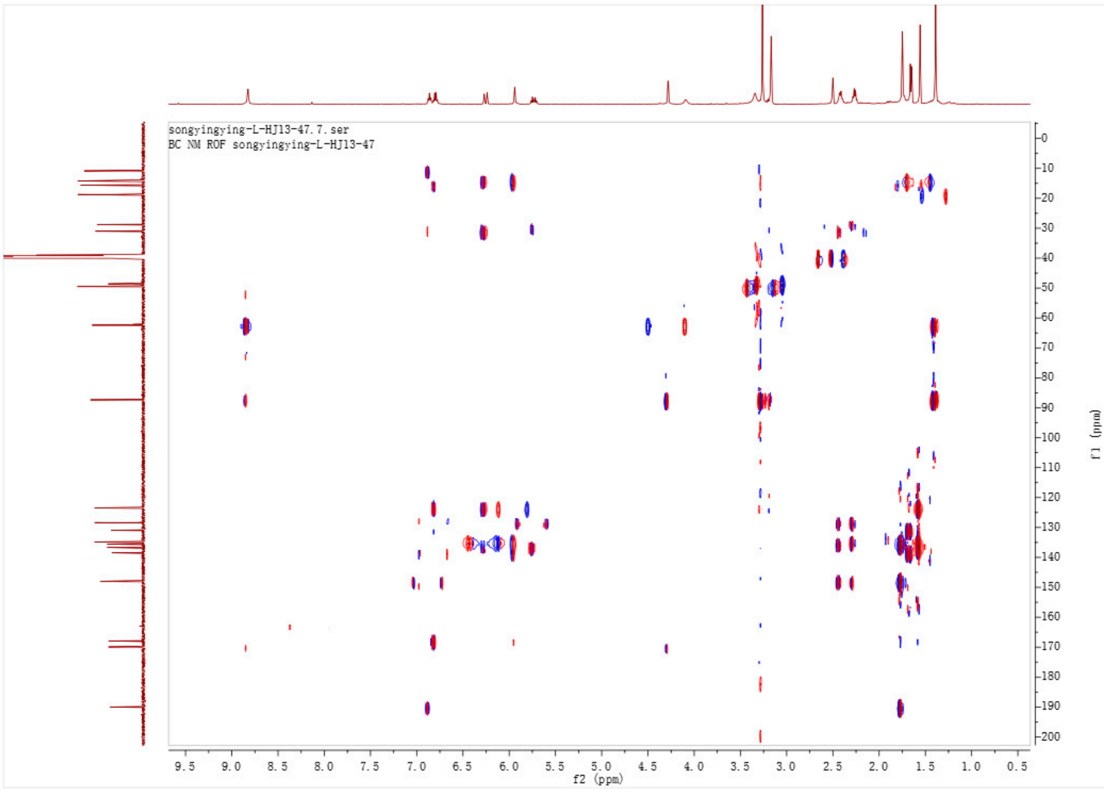

Figure S6 HMBC spectrum of compound **1** in DMSO-d_6

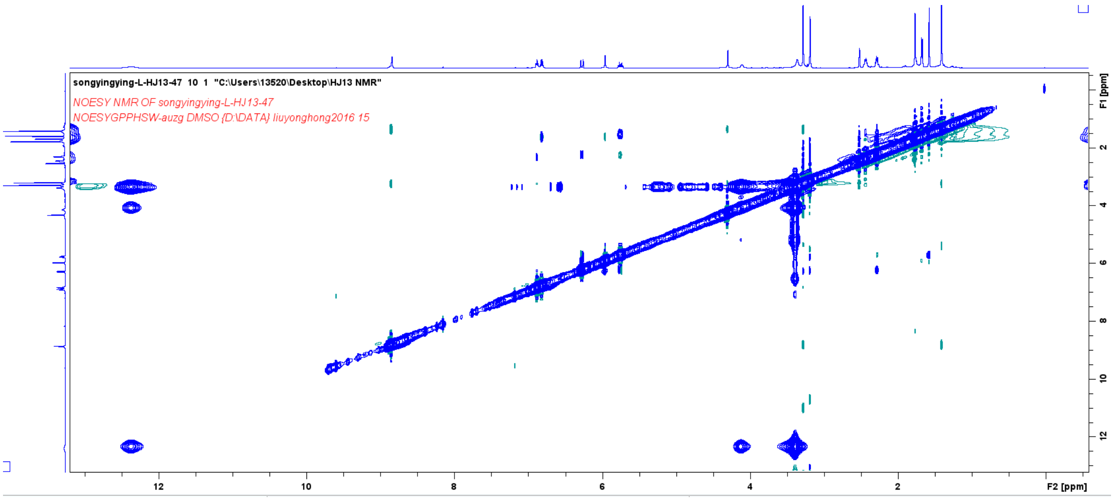

Figure S7 NOESY spectrum of compound **1** in DMSO-d_6

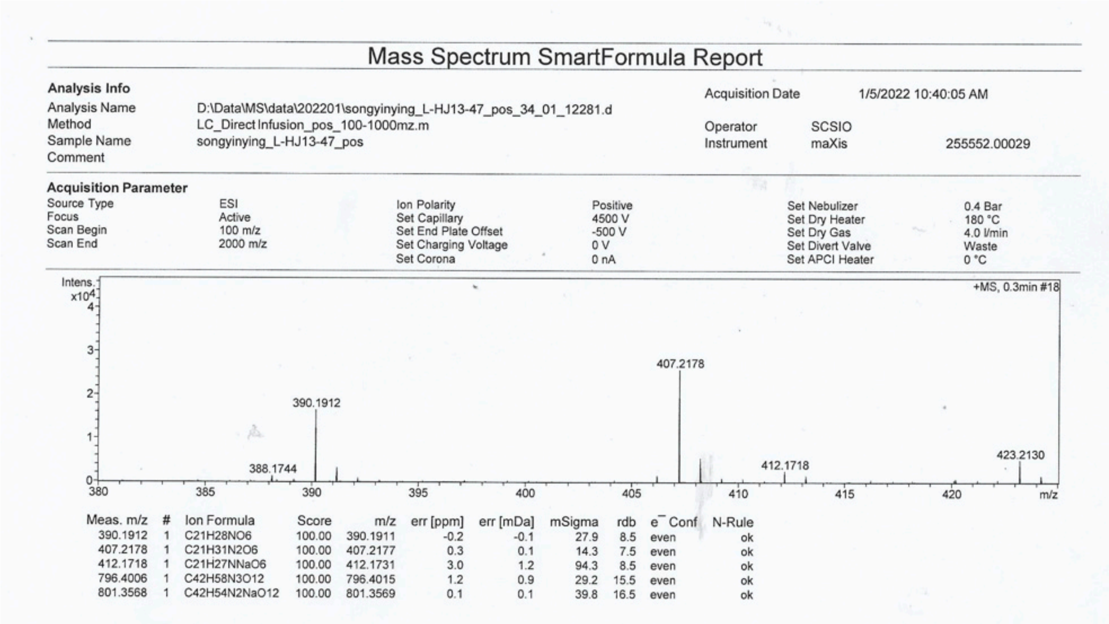

Mass Spectrum SmartFormula Report

Analysis Info

Analysis Name D:\Data\MS\data\202201\songyinying_L-HJ13-47_pos_34_01_12281.d
Method LC_Direct Infusion_pos_100-1000mz.m
Sample Name songyinying_L-HJ13-47_pos
Comment

Acquisition Date 1/5/2022 10:40:05 AM
Operator SCSIO
Instrument maXis 255552.00029

Acquisition Parameter

Source Type	ESI	Ion Polarity	Positive	Set Nebulizer	0.4 Bar
Focus	Active	Set Capillary	4500 V	Set Dry Heater	180 °C
Scan Begin	100 m/z	Set End Plate Offset	-500 V	Set Dry Gas	4.0 l/min
Scan End	2000 m/z	Set Charging Voltage	0 V	Set Divert Valve	Waste
		Set Corona	0 nA	Set APCI Heater	0 °C

Meas. m/z	#	Ion Formula	Score	m/z	err [ppm]	err [mDa]	mSigma	rdb	e⁻ Conf	N-Rule
390.1912	1	C21H28NO6	100.00	390.1911	-0.2	-0.1	27.9	8.5	even	ok
407.2178	1	C21H31N2O6	100.00	407.2177	0.3	0.1	14.3	7.5	even	ok
412.1718	1	C21H27NNaO6	100.00	412.1731	3.0	1.2	94.3	8.5	even	ok
796.4006	1	C42H58N3O12	100.00	796.4015	1.2	0.9	29.2	15.5	even	ok
801.3568	1	C42H54N2NaO12	100.00	801.3569	0.1	0.1	39.8	16.5	even	ok

Figure S8 HRESIMS spectrum of compound **1**

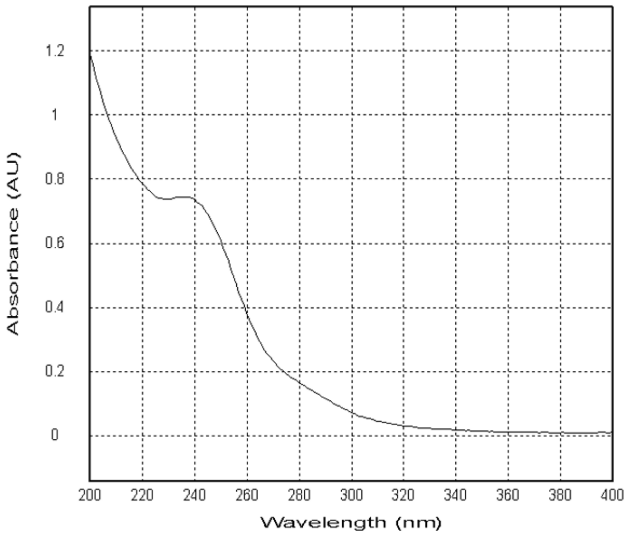

Figure S9 UV spectrum of **1**

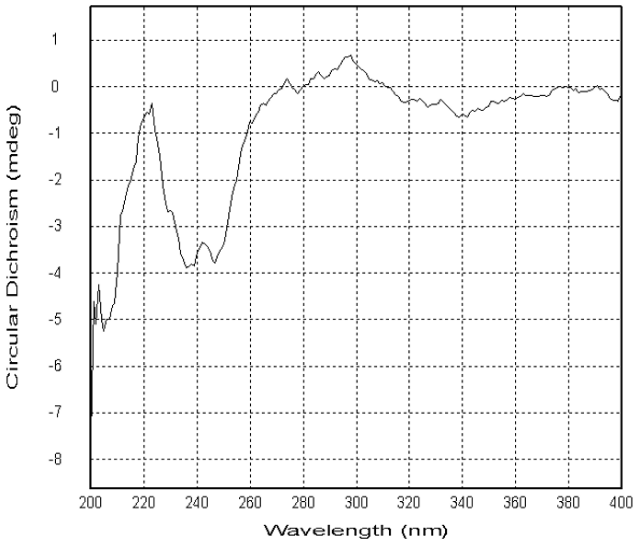

Figure S10 CD spectrum of **1**

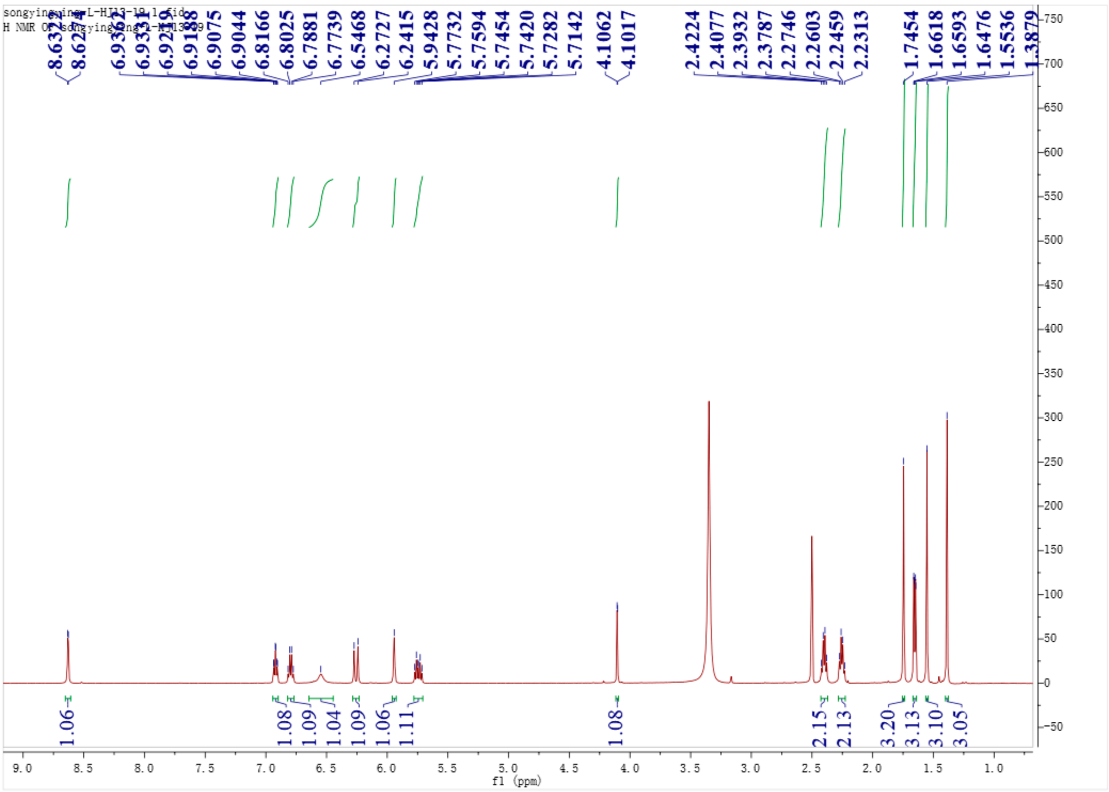

Figure S11 ¹H NMR spectrum of compound **2** in DMSO-d_6

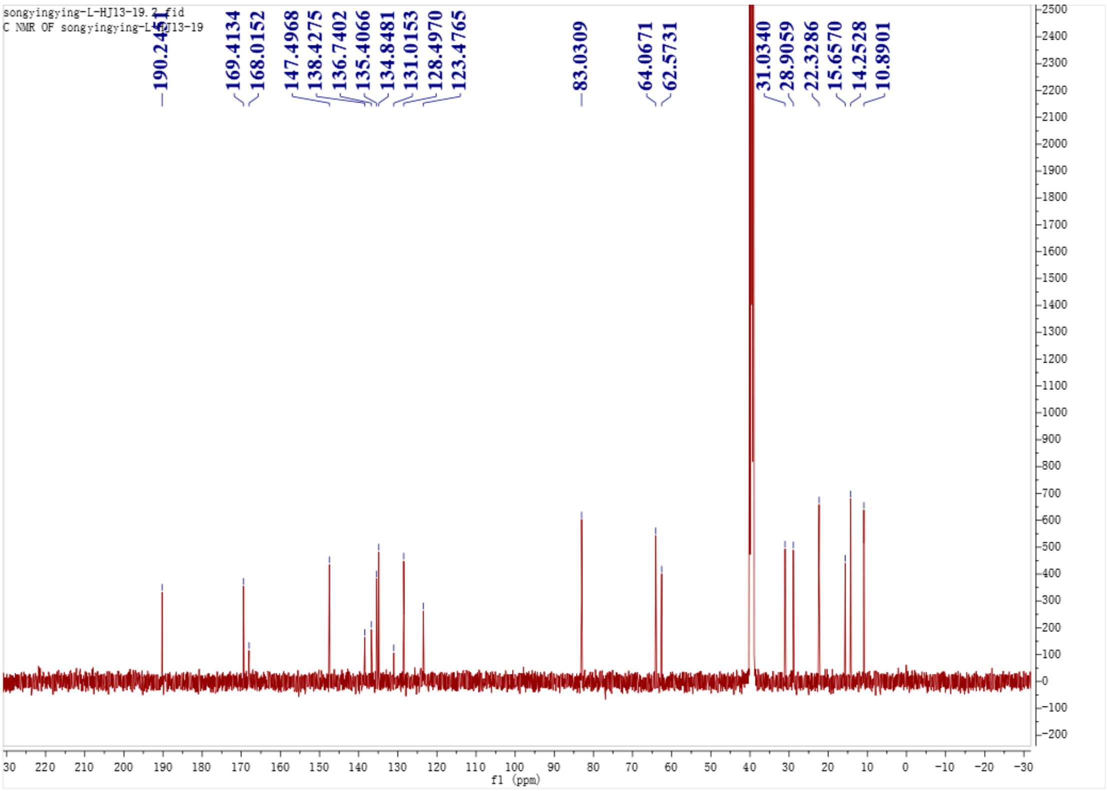

Figure S12 ¹³C NMR spectrum of compound **2** in DMSO-d_6

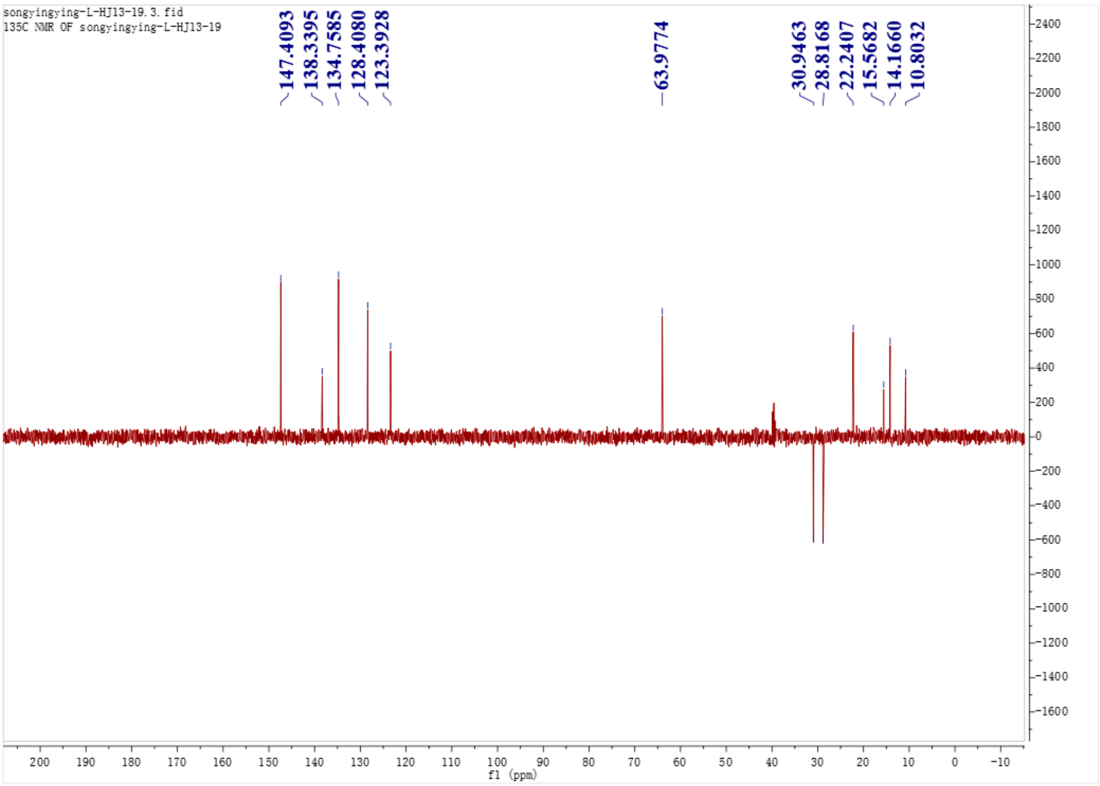

Figure S13 DEPT135 spectrum of compound **2** in DMSO-d_6

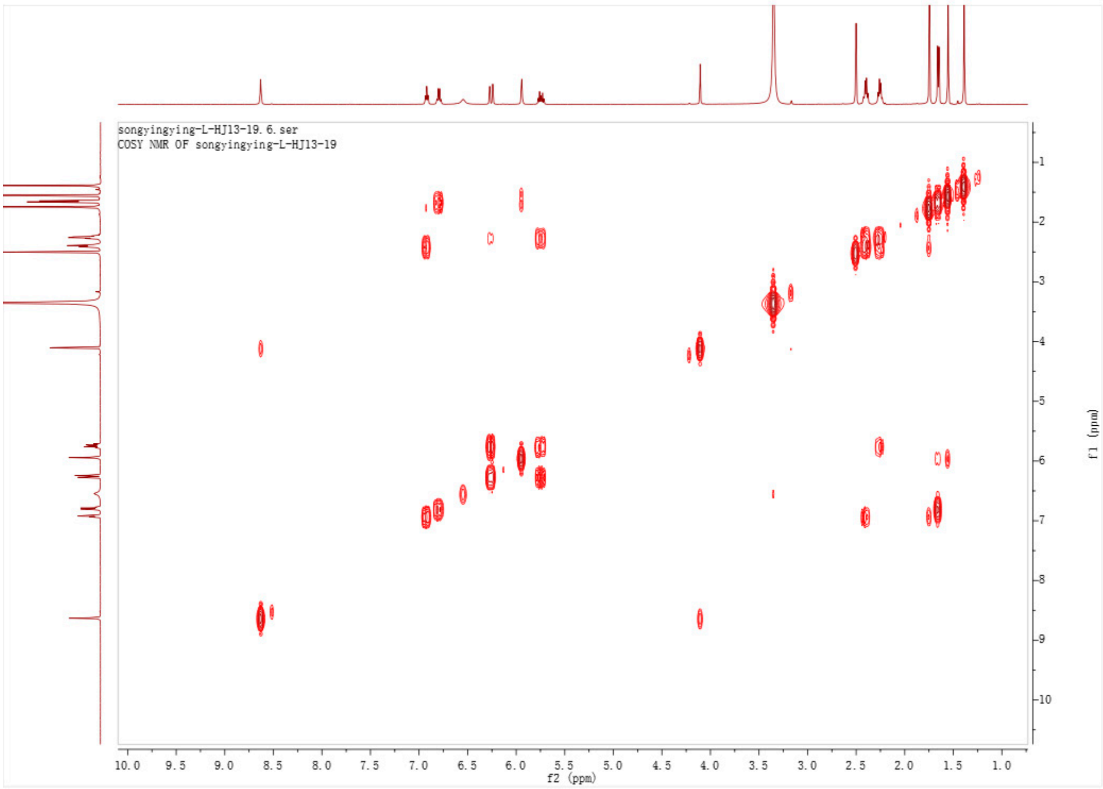

Figure S14 ^{1}H-^{1}H COSY spectrum of compound **2** in DMSO-d_6

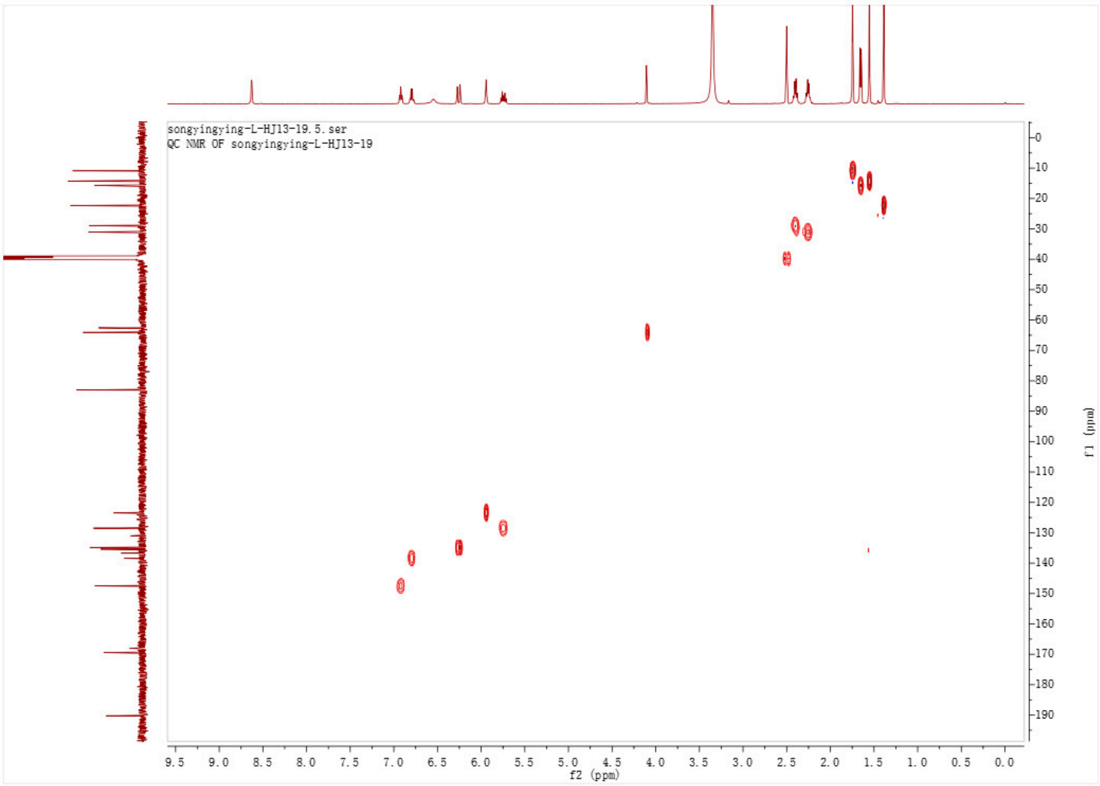

Figure S15 HSQC spectrum of compound **2** in DMSO-d_6

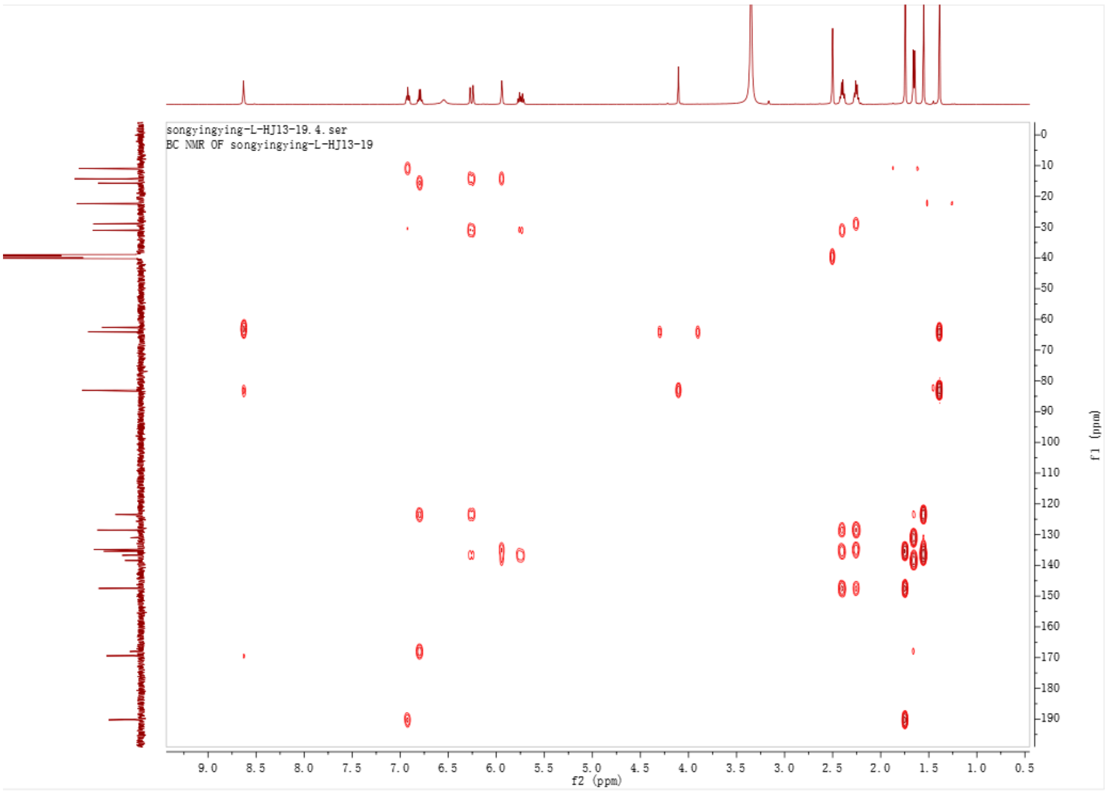

Figure S16 HMBC spectrum of compound **2** in DMSO-d_6

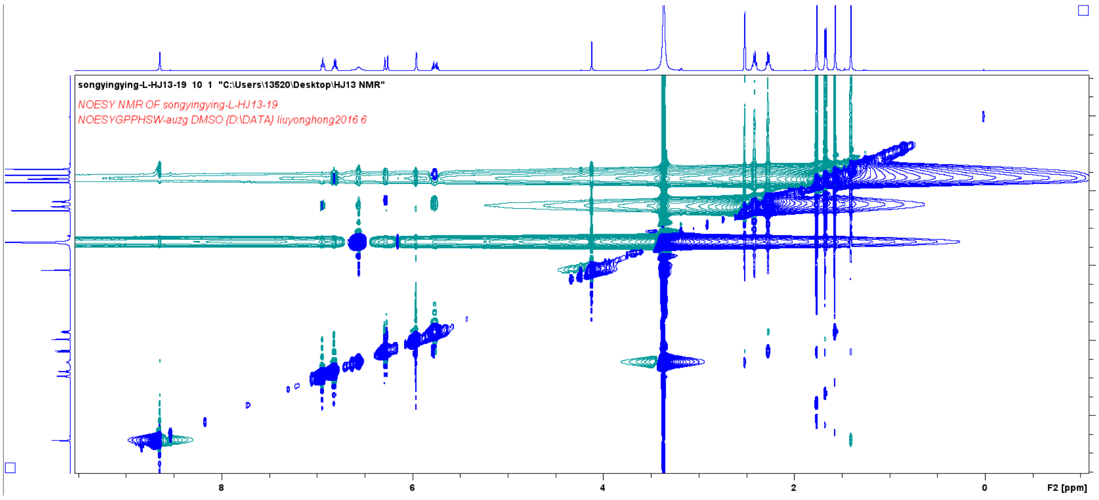

Figure S17 NOESY spectrum of compound **2** in DMSO-d_6

Mass Spectrum SmartFormula Report

Analysis Info

Analysis Name	D:\Data\MS\data\202108\songyingying_L-HJ13-19_neg_12_01_11363.d	Acquisition Date	8/24/2021 9:09:47 AM
Method	LC_Direct Infusion_neg_70-500mz.m	Operator	SCSIO
Sample Name	songyingying_L-HJ13-19_neg	Instrument	maXis 255552.00029
Comment			

Acquisition Parameter

Source Type	ESI	Ion Polarity	Negative	Set Nebulizer	0.4 Bar
Focus	Active	Set Capillary	4000 V	Set Dry Heater	180 °C
Scan Begin	100 m/z	Set End Plate Offset	-500 V	Set Dry Gas	4.0 l/min
Scan End	1500 m/z	Set Charging Voltage	0 V	Set Divert Valve	Waste
		Set Corona	0 nA	Set APCI Heater	0 °C

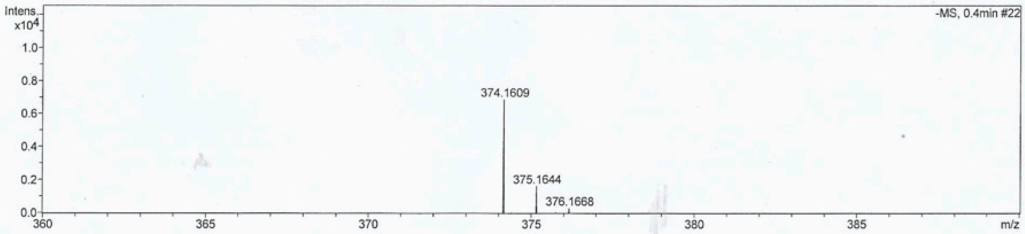

Meas. m/z	#	Ion Formula	Score	m/z	err [ppm]	err [mDa]	mSigma	rdb	e⁻ Conf	N-Rule
374.1609	1	C20H24NO6	100.00	374.1609	0.1	0.0	8.8	9.5	even	ok
749.3300	1	C40H49N2O12	100.00	749.3291	-1.3	-0.9	30.7	17.5	even	ok

Figure S18 HRESIMS spectrum of compound **2**

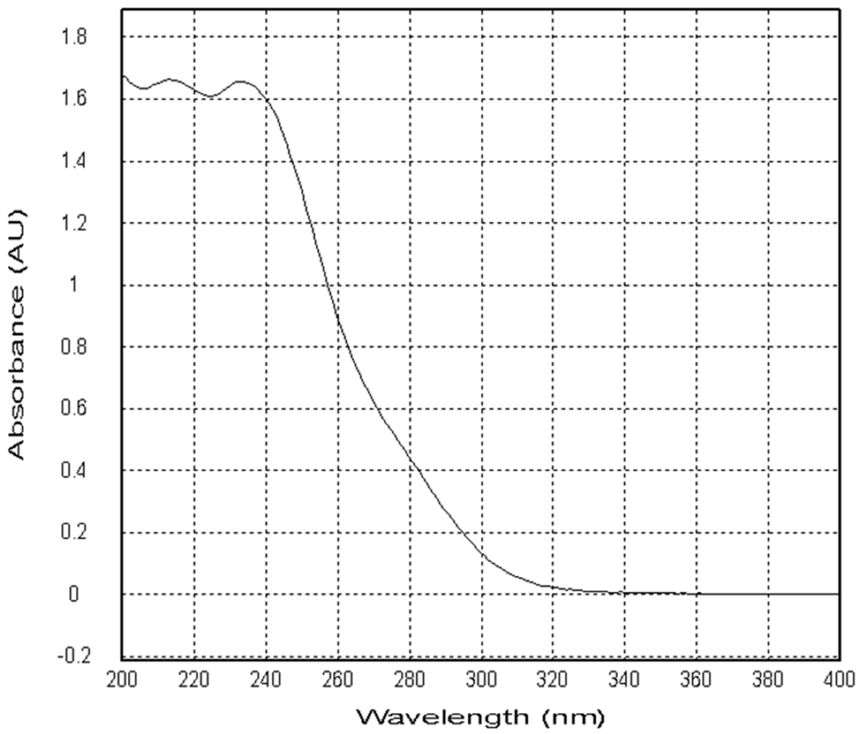

Figure S19 UV spectrum of **2**

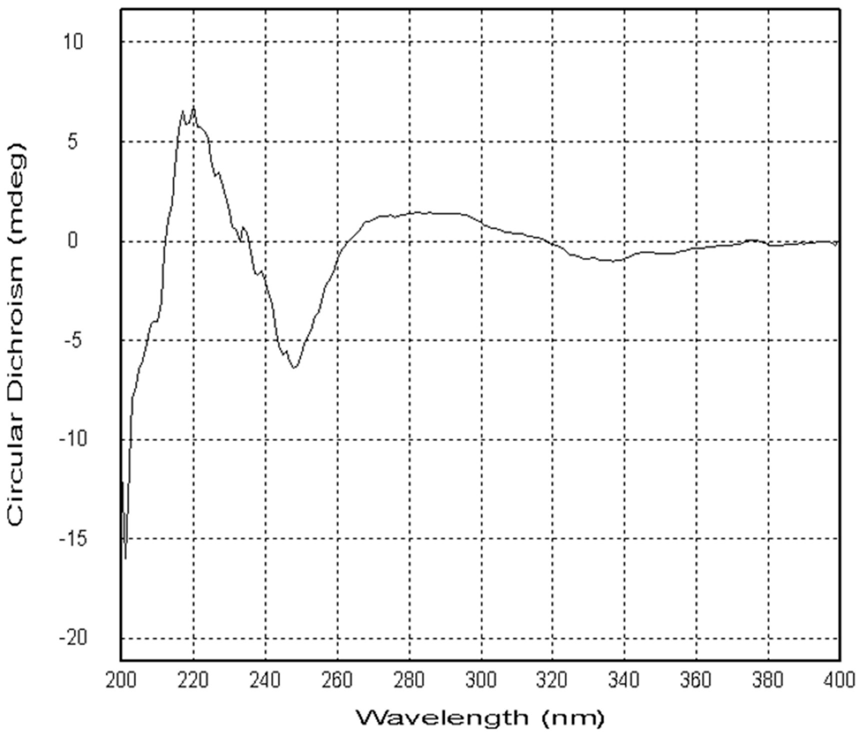

Figure S20 CD spectrum of **2**

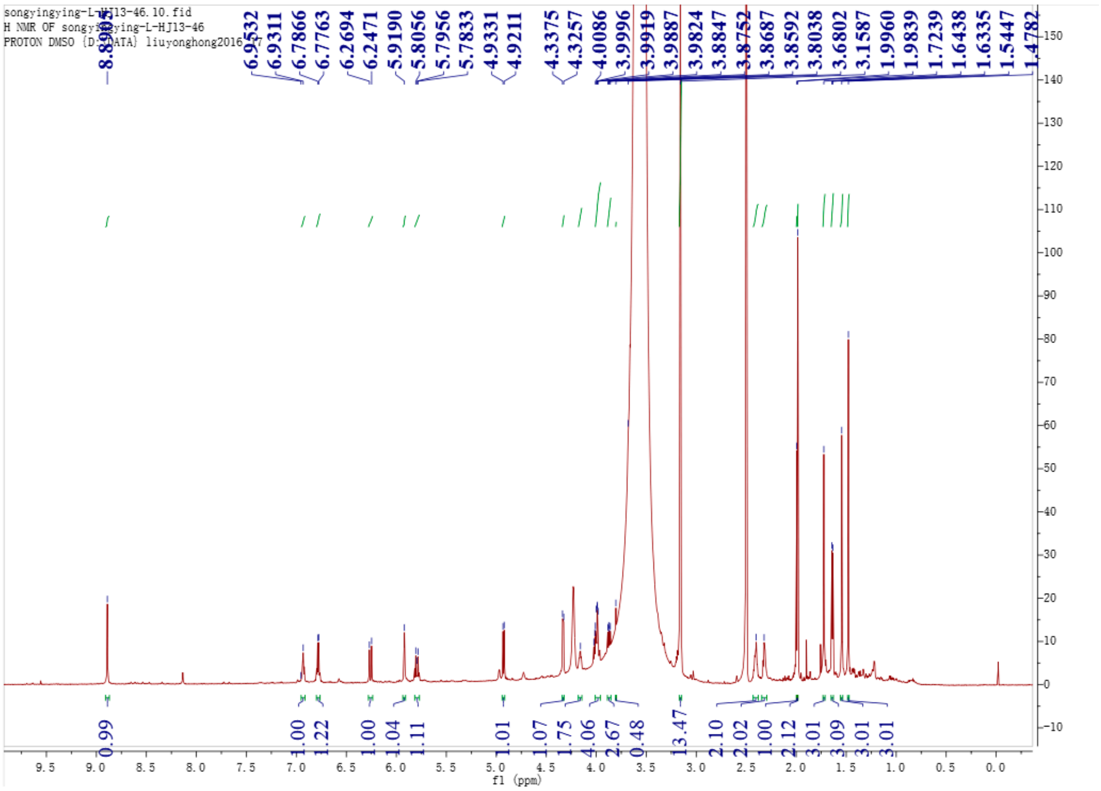

Figure S21 ^{1}H NMR spectrum of compound **3** in DMSO-d_6

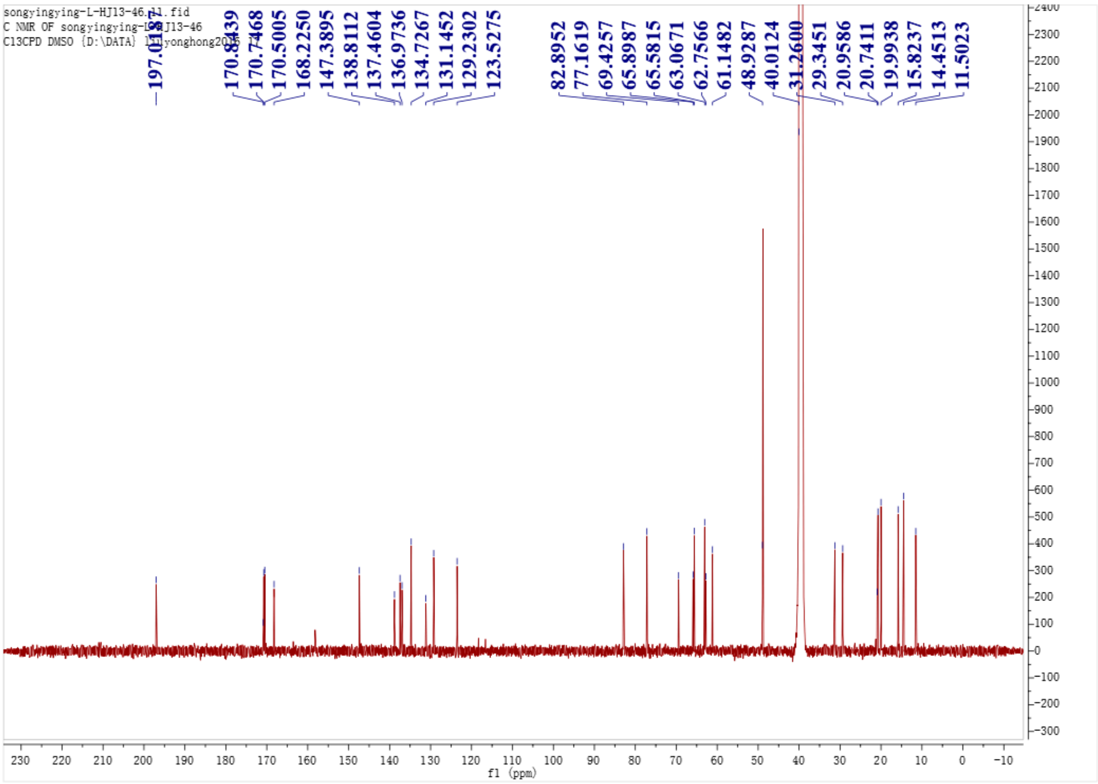

Figure S22 ^{13}C NMR spectrum of compound **3** in DMSO-d_6

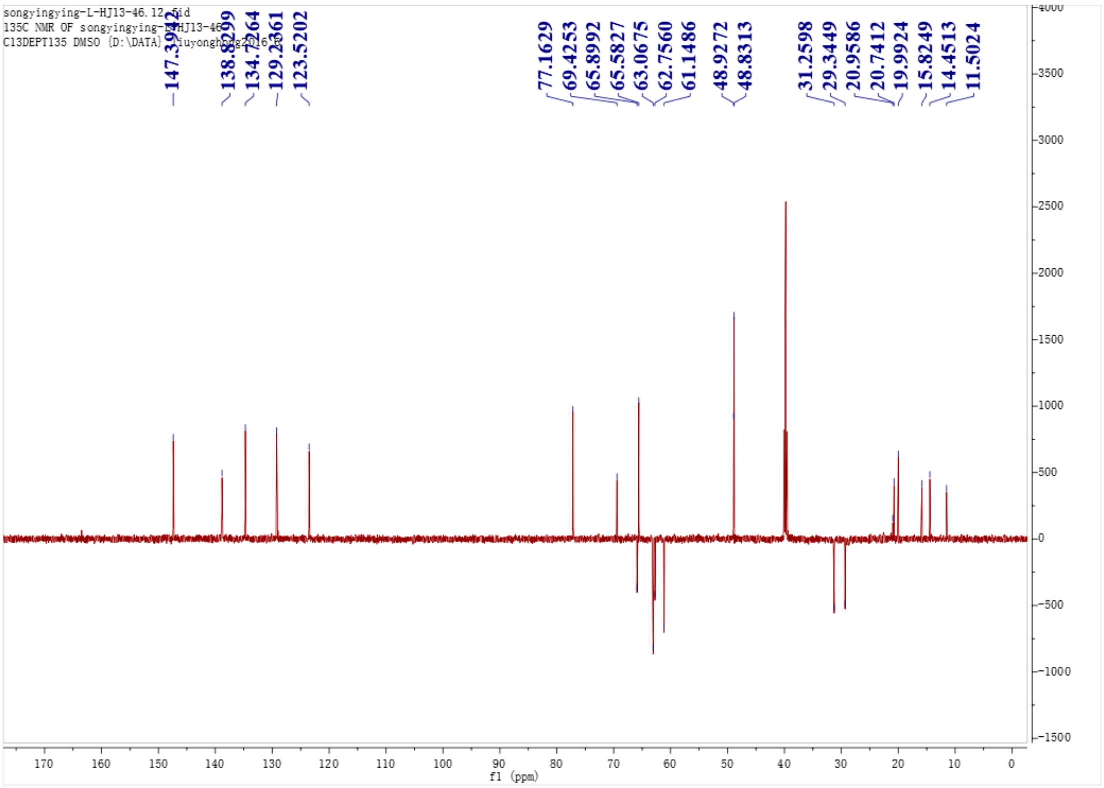

Figure S23 DEPT135 spectrum of compound **3** in DMSO-d_6

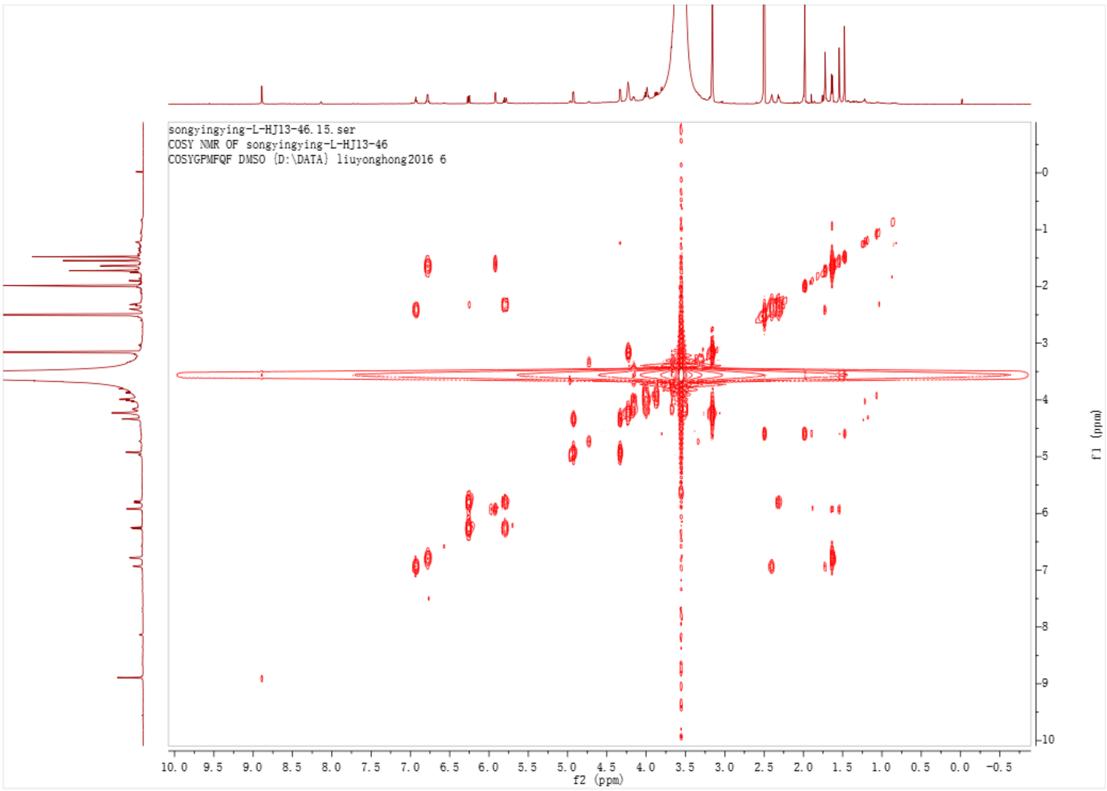

Figure S24 ^{1}H-^{1}H COSY spectrum of compound **3** in DMSO-d_6

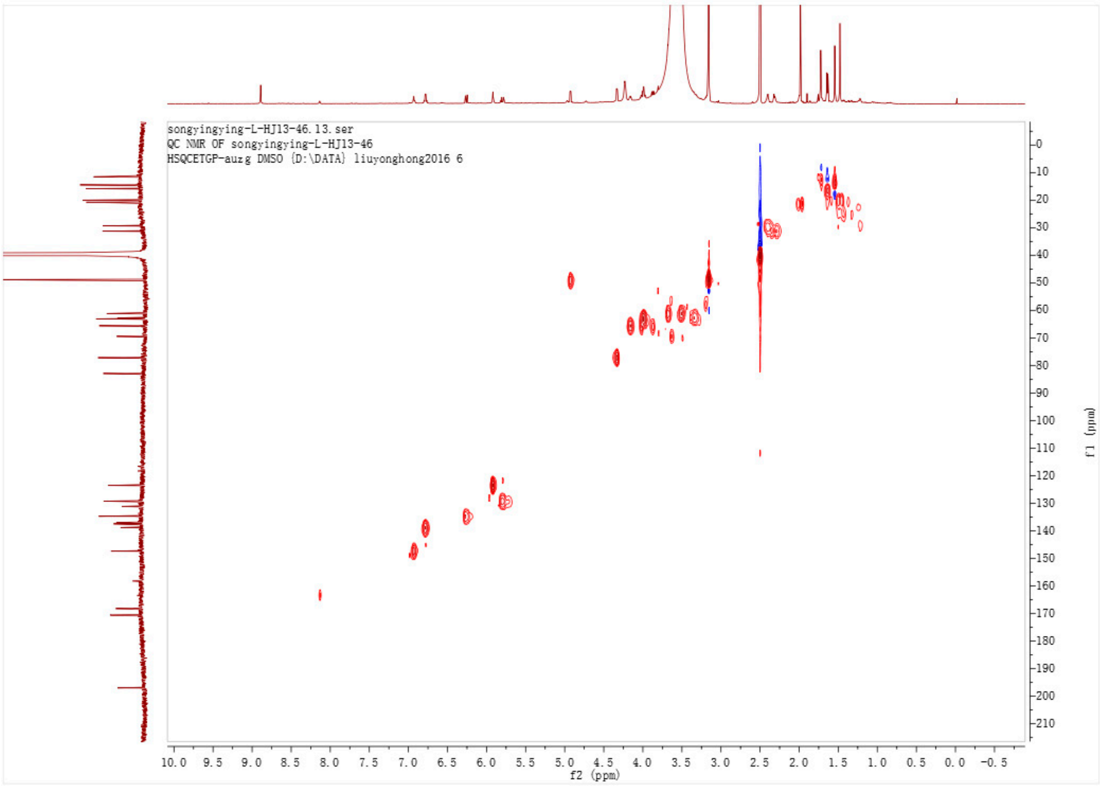

Figure S25 HSQC spectrum of compound **3** in DMSO-d_6

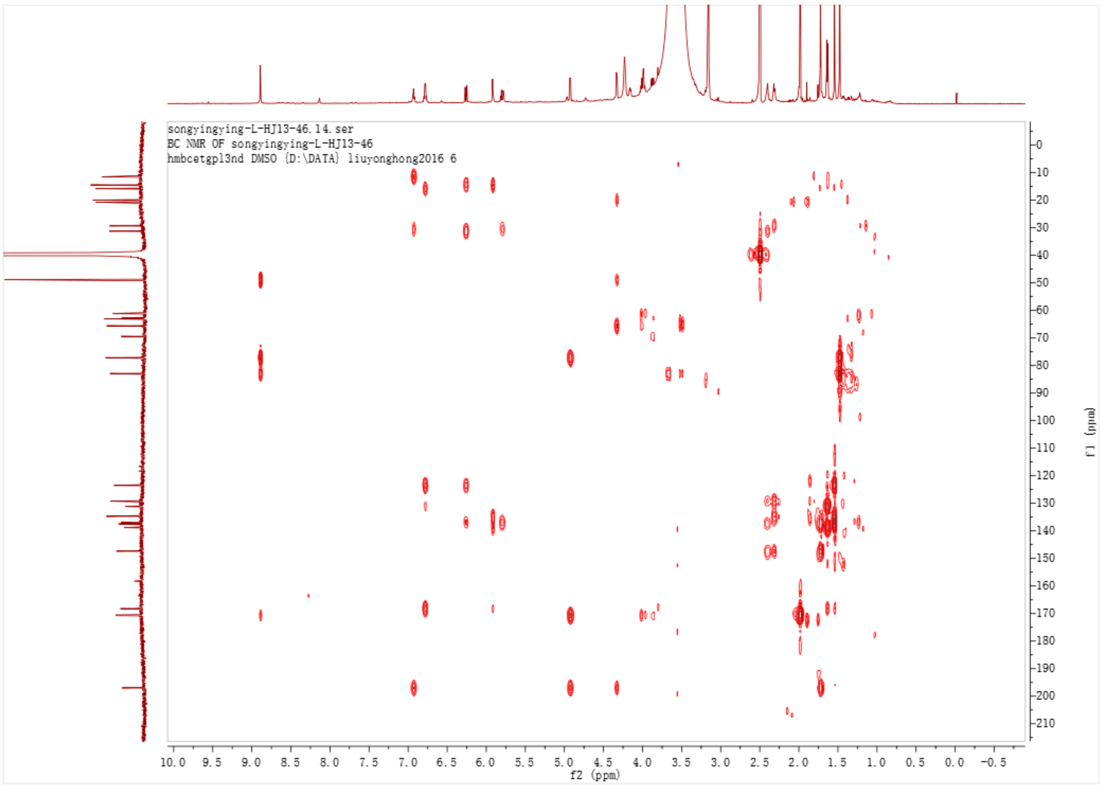

Figure S26 HMBC spectrum of compound **3** in DMSO-d_6

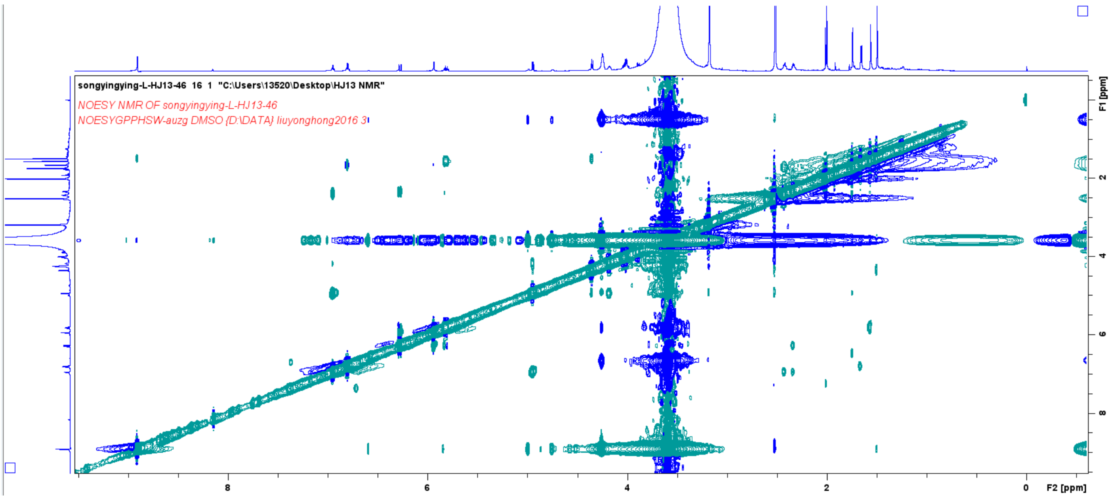

Figure S27 NOESY spectrum of compound **3** in DMSO-d_6

Mass Spectrum SmartFormula Report

Analysis Info

Analysis Name	D:\Data\MS\data\202203\songyingying_L-HJ13-46_pos_4_01_12834.d	Acquisition Date	3/18/2022 9:23:05 AM
Method	LC_Direct Infusion_pos_70-500mz.m	Operator	SCSIO
Sample Name	songyingying_L-HJ13-46_pos	Instrument	maXis 255552.00029
Comment			

Acquisition Parameter

Source Type	ESI	Ion Polarity	Positive	Set Nebulizer	0.4 Bar
Focus	Active	Set Capillary	4500 V	Set Dry Heater	180 °C
Scan Begin	70 m/z	Set End Plate Offset	-500 V	Set Dry Gas	4.0 l/min
Scan End	1500 m/z	Set Charging Voltage	0 V	Set Divert Valve	Waste
		Set Corona	0 nA	Set APCI Heater	0 °C

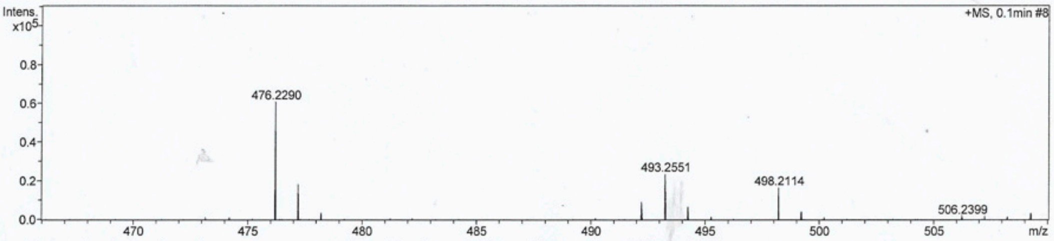

Meas. m/z	#	Ion Formula	Score	m/z	err [ppm]	err [mDa]	mSigma	rdb	e⁻ Conf	N-Rule
476.2290	1	C25H34NO8	100.00	476.2279	2.2	1.1	9.6	9.5	even	ok
493.2551	1	C25H37N2O8	100.00	493.2544	1.4	0.7	6.1	8.5	even	ok
498.2114	1	C25H33NNaO8	100.00	498.2098	-3.1	-1.5	11.5	9.5	even	ok
951.4510	1	C50H67N2O16	100.00	951.4485	2.6	2.4	38.3	18.5	even	ok
968.4776	1	C50H70N3O16	100.00	968.4751	-2.6	-2.5	7.0	17.5	even	ok
973.4333	1	C50H66N2NaO16	100.00	973.4305	-2.9	-2.8	27.8	18.5	even	ok

Figure S28 HRESIMS spectrum of compound **3**

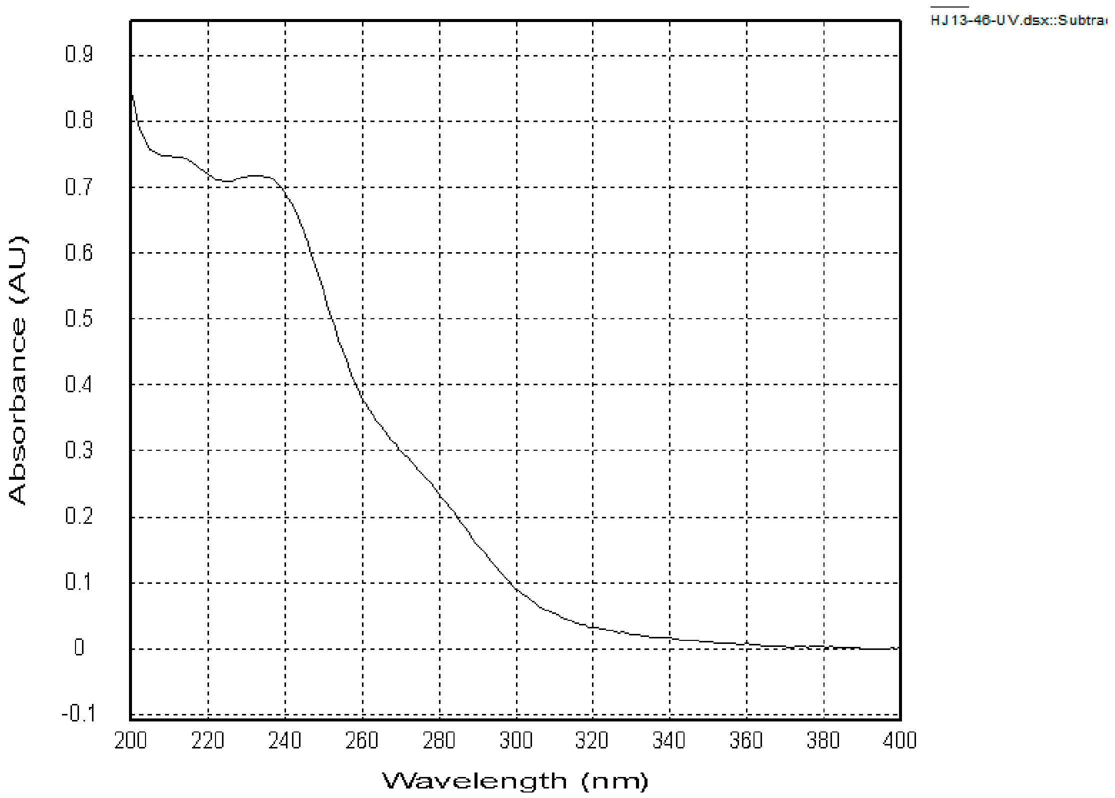

Figure S29 UV spectrum of **3**

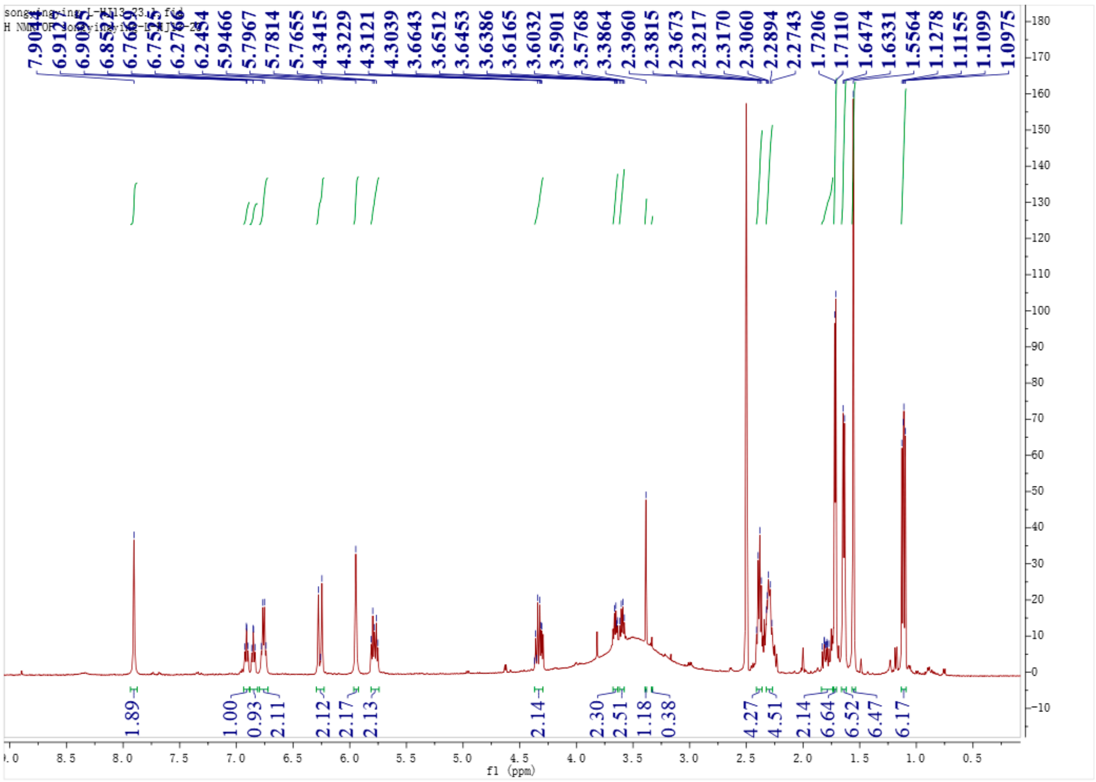

Figure S30 ^{1}H NMR spectrum of compound **4** in DMSO-d_6

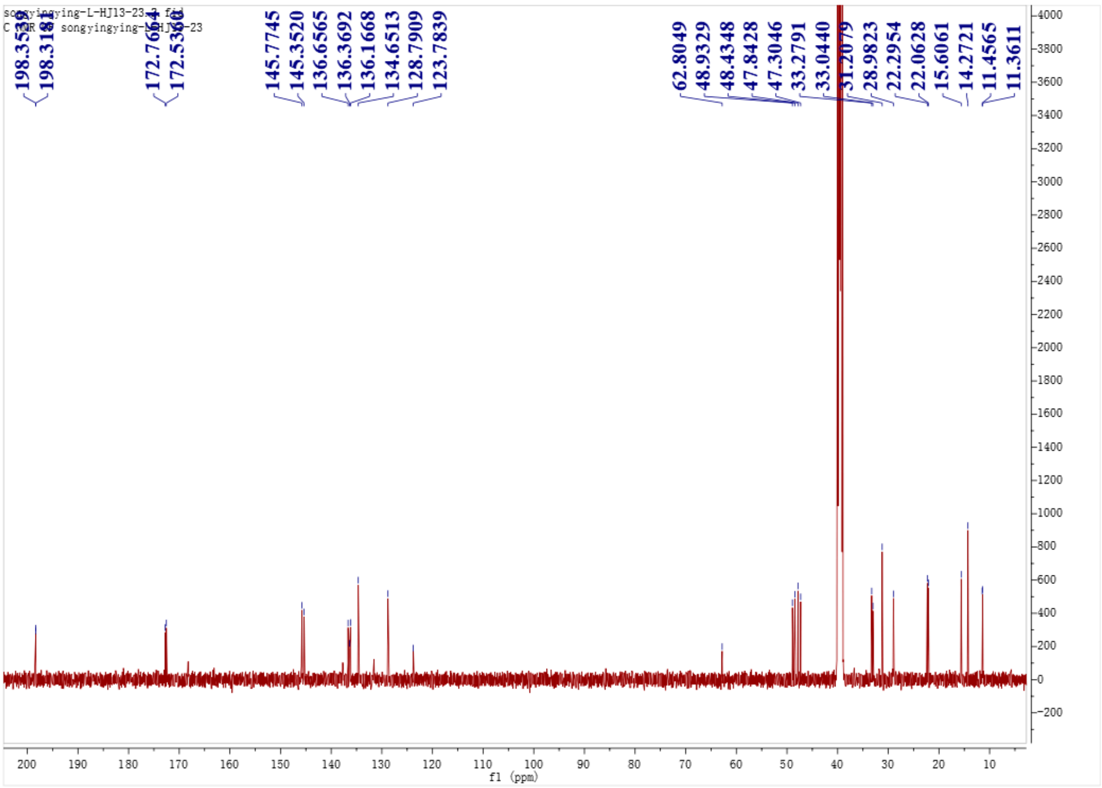

Figure S31 ^{13}C NMR spectrum of compound **4** in DMSO-d_6

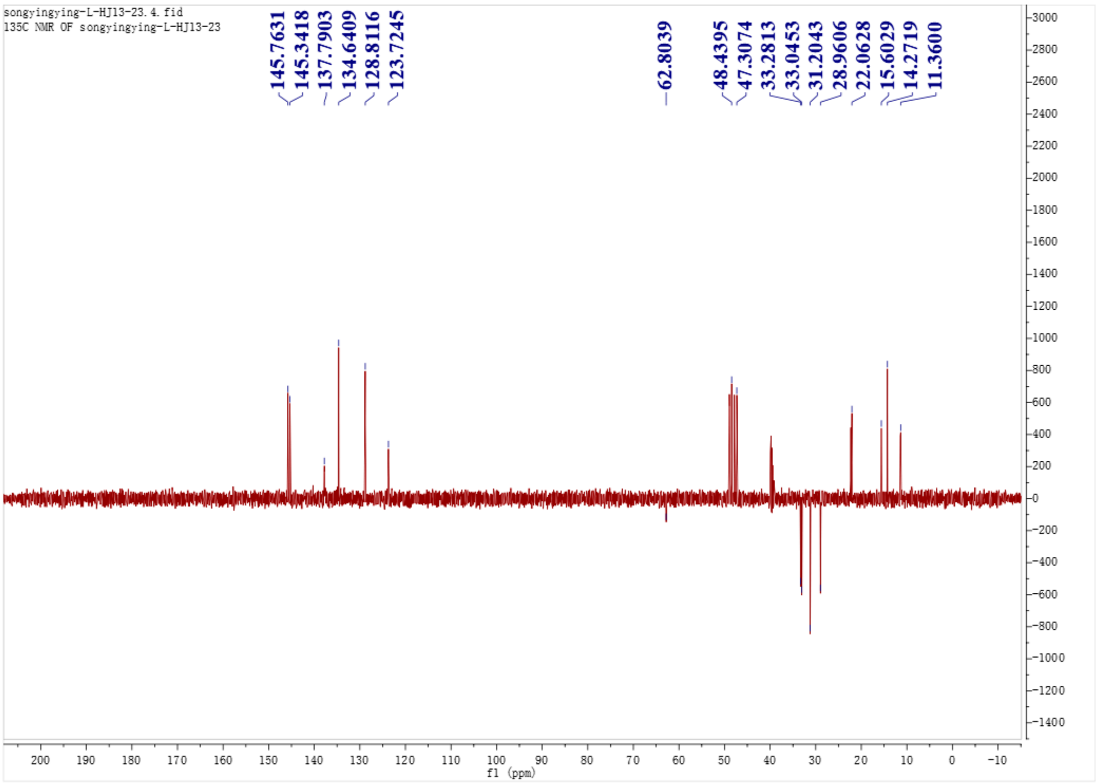

Figure S32 DEPT135 spectrum of compound **4** in DMSO-d_6

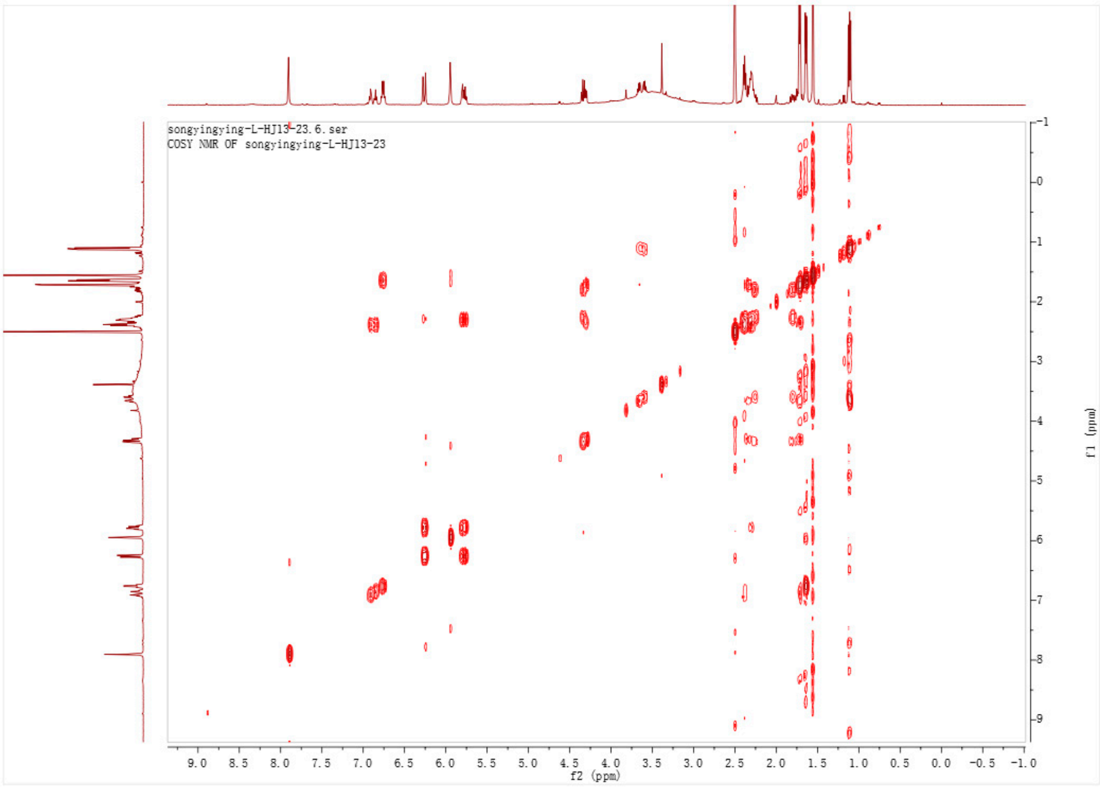

Figure S33 ^{1}H-^{1}H COSY spectrum of compound **4** in DMSO-d_6

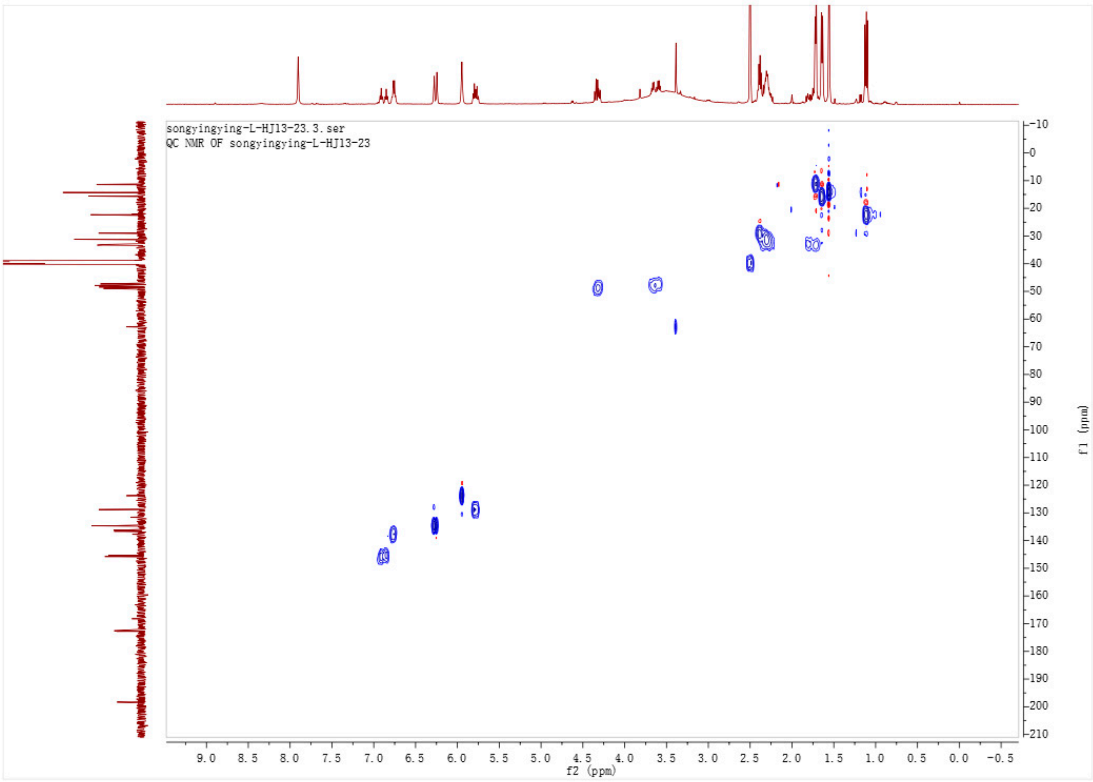

Figure S34 HSQC spectrum of compound **4** in DMSO-d_6

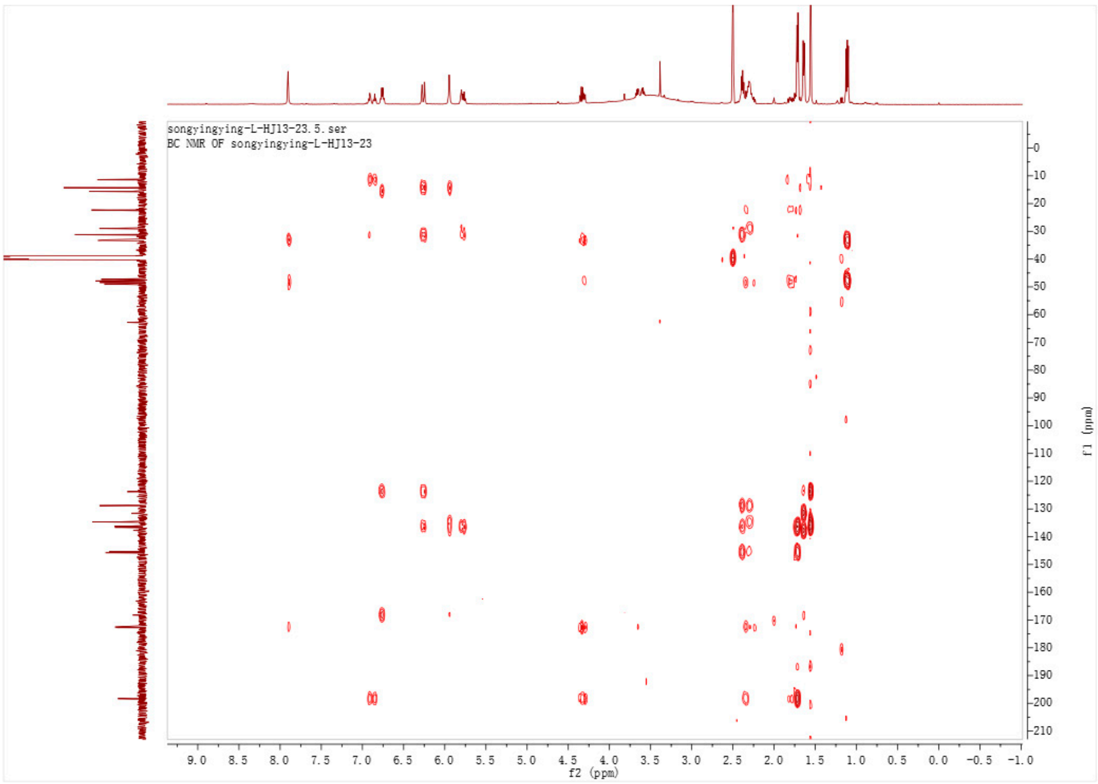

Figure S35 HMBC spectrum of compound **4** in DMSO-d_6

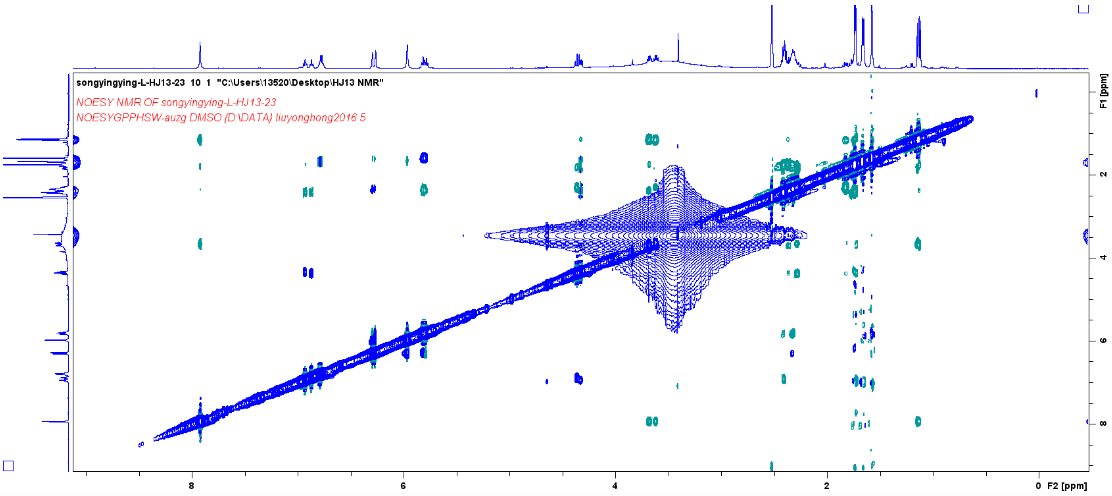

Figure S36 NOESY spectrum of compound **4** in DMSO-d_6

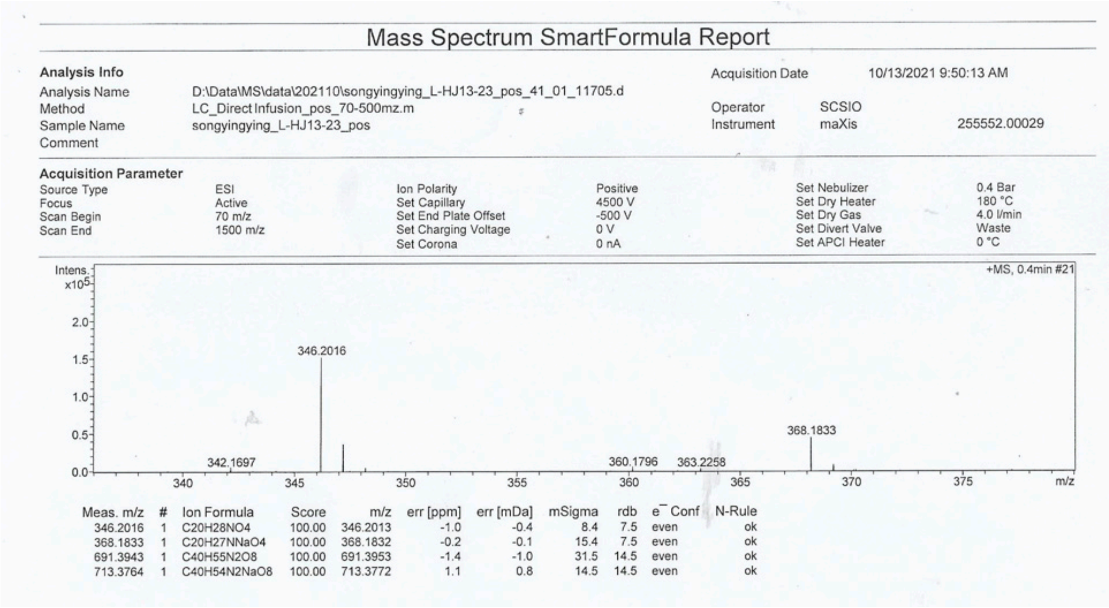

Figure S37 HRESIMS spectrum of compound **4**

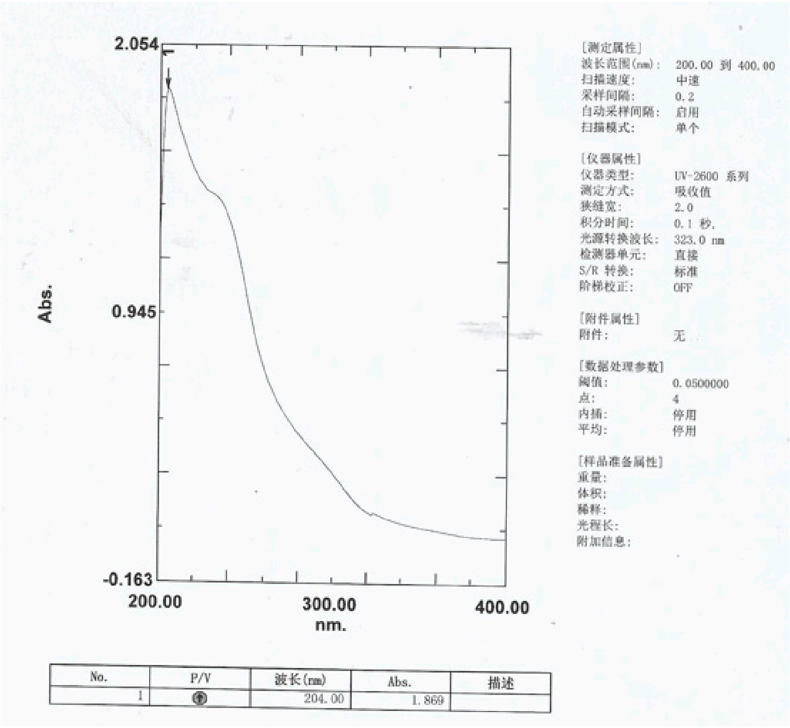

Figure S38 UV spectrum of **4**

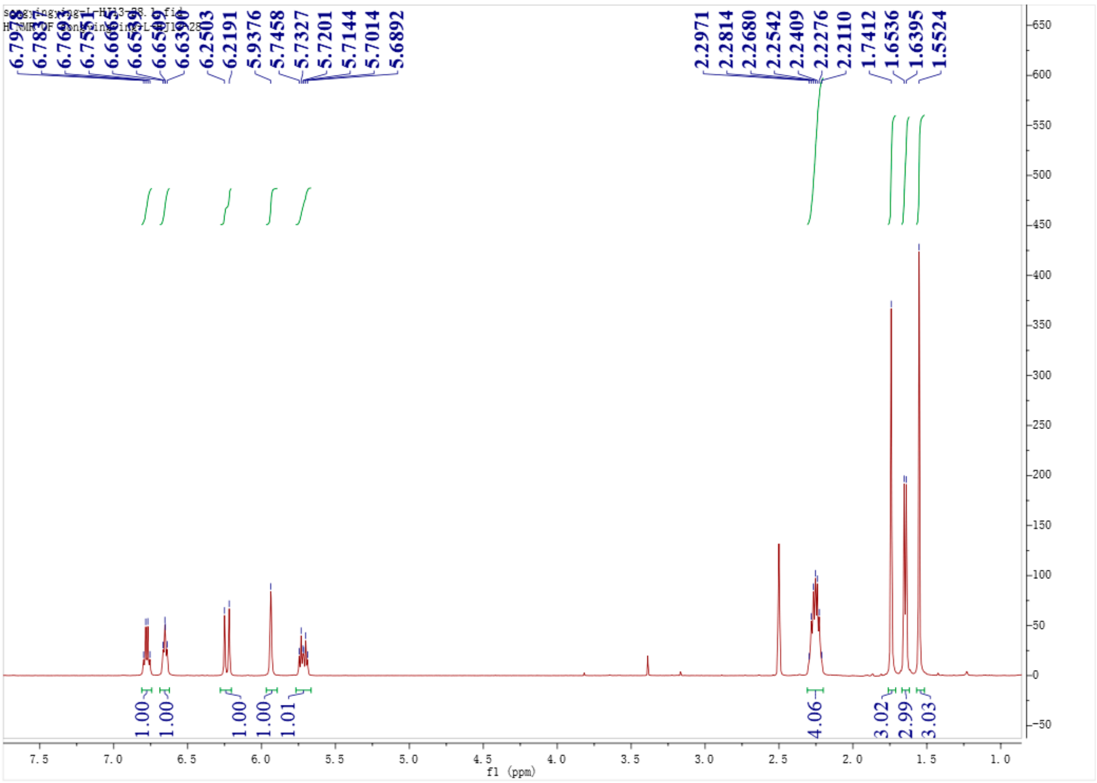

Figure S39 ^{1}H NMR spectrum of compound **5** in DMSO-d_6

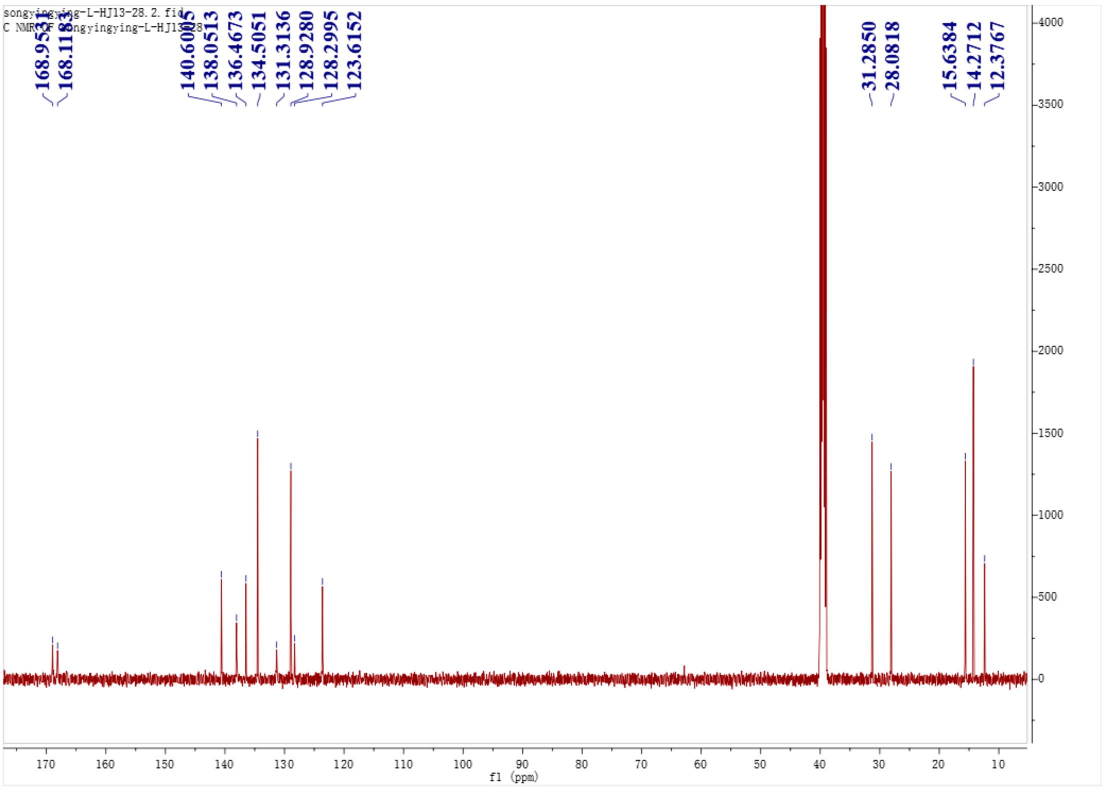

Figure S40 ^{13}C NMR spectrum of compound **5** in DMSO-d_6

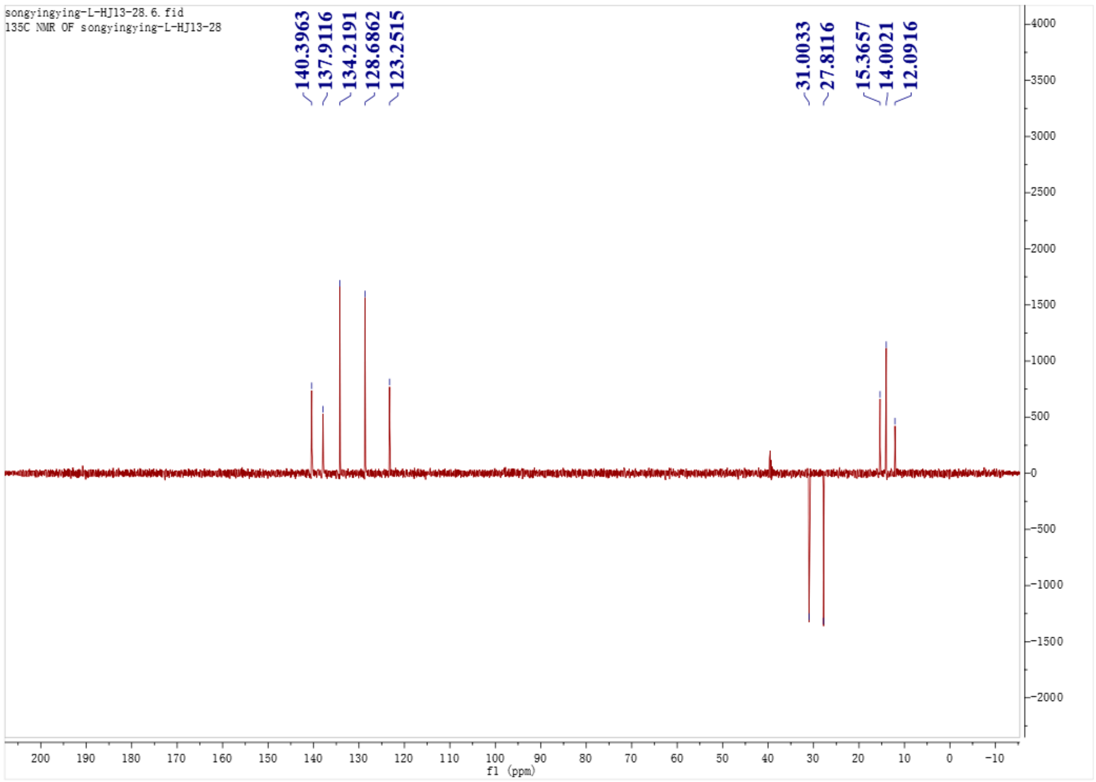

Figure S41 DEPT135 spectrum of compound **5** in DMSO-d_6

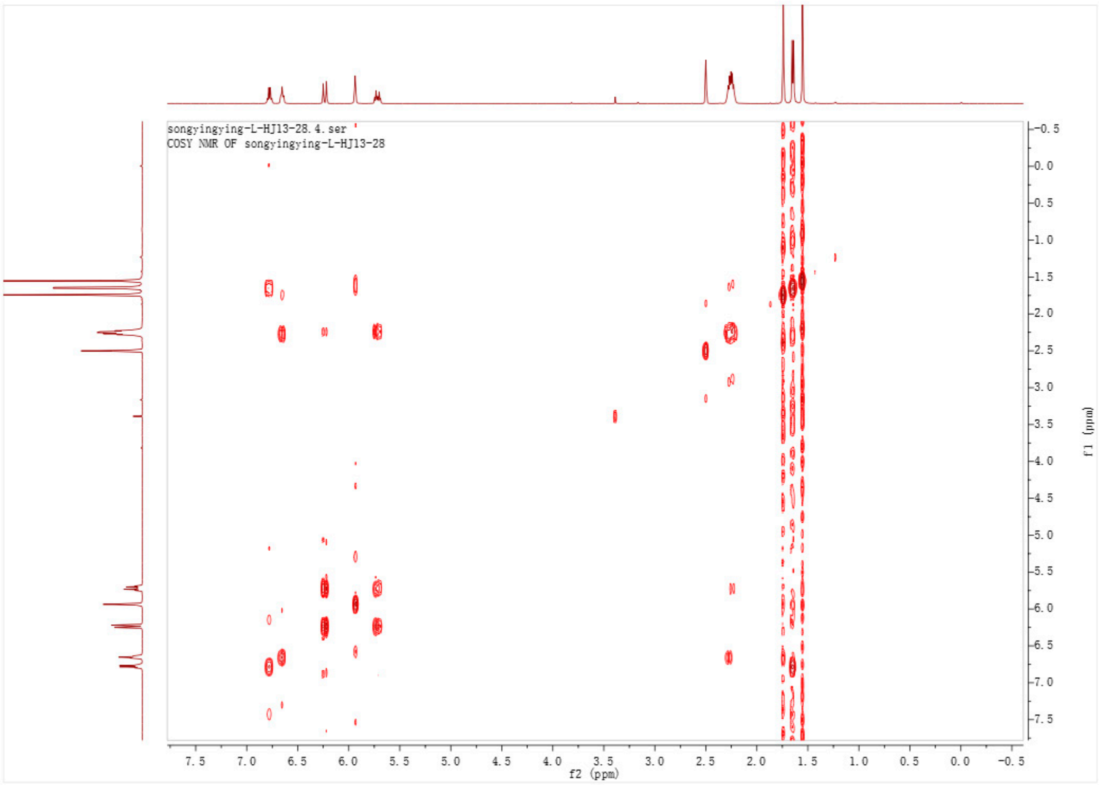

Figure S42 ^{1}H-^{1}H COSY spectrum of compound **5** in DMSO-d_6

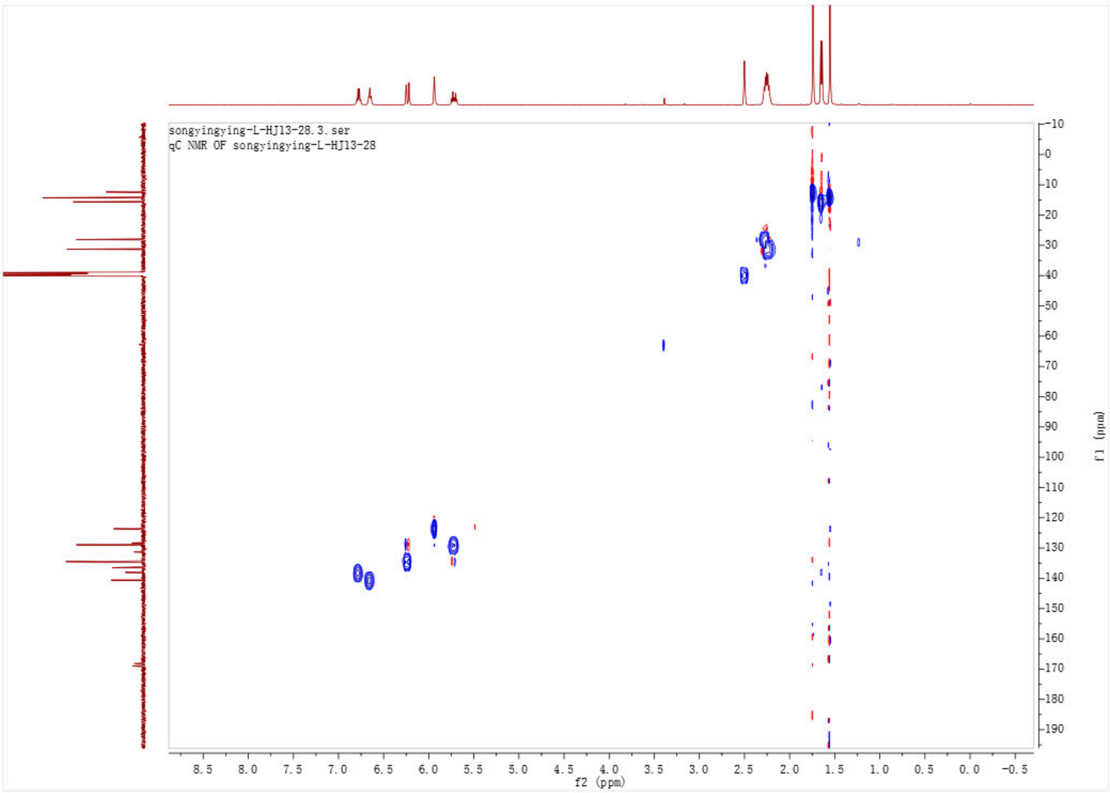

Figure S43 HSQC spectrum of compound **5** in DMSO-d_6

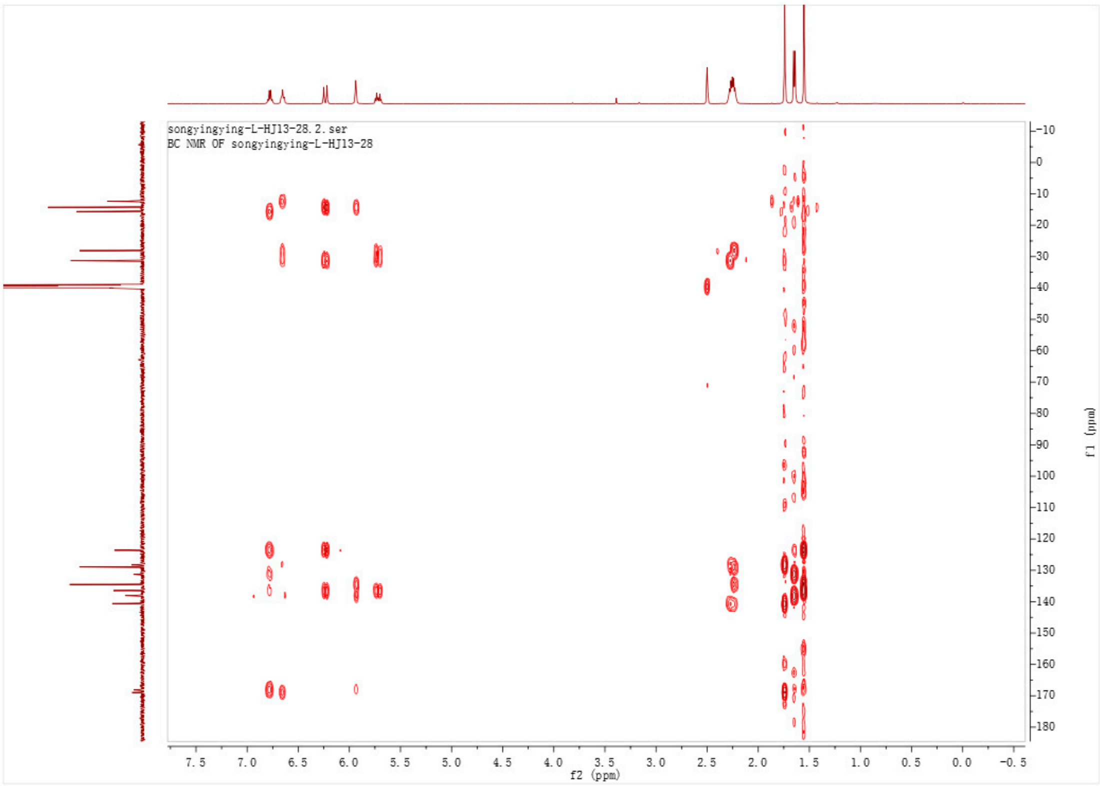

Figure S44 HMBC spectrum of compound **5** in DMSO-d_6

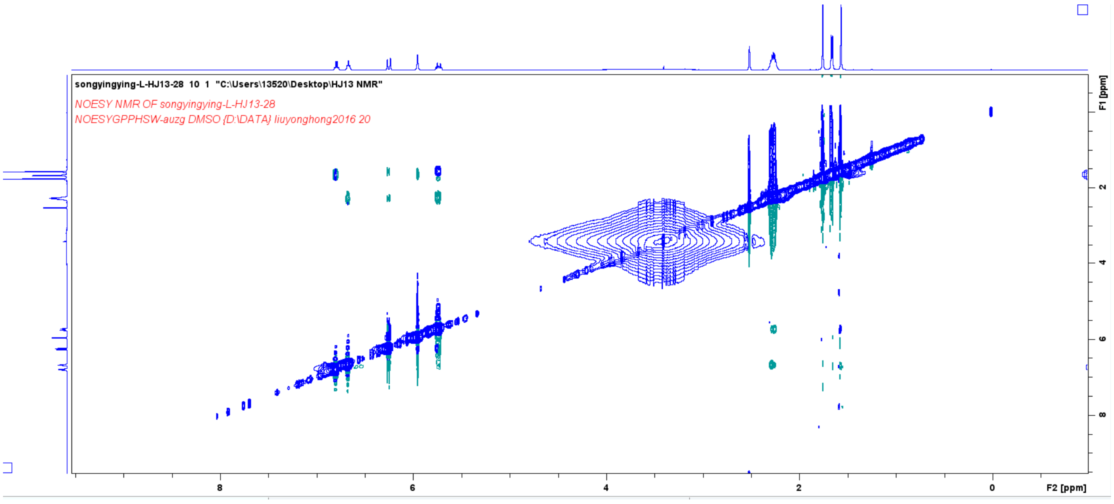

Figure S45 NOESY spectrum of compound **5** in DMSO-d_6

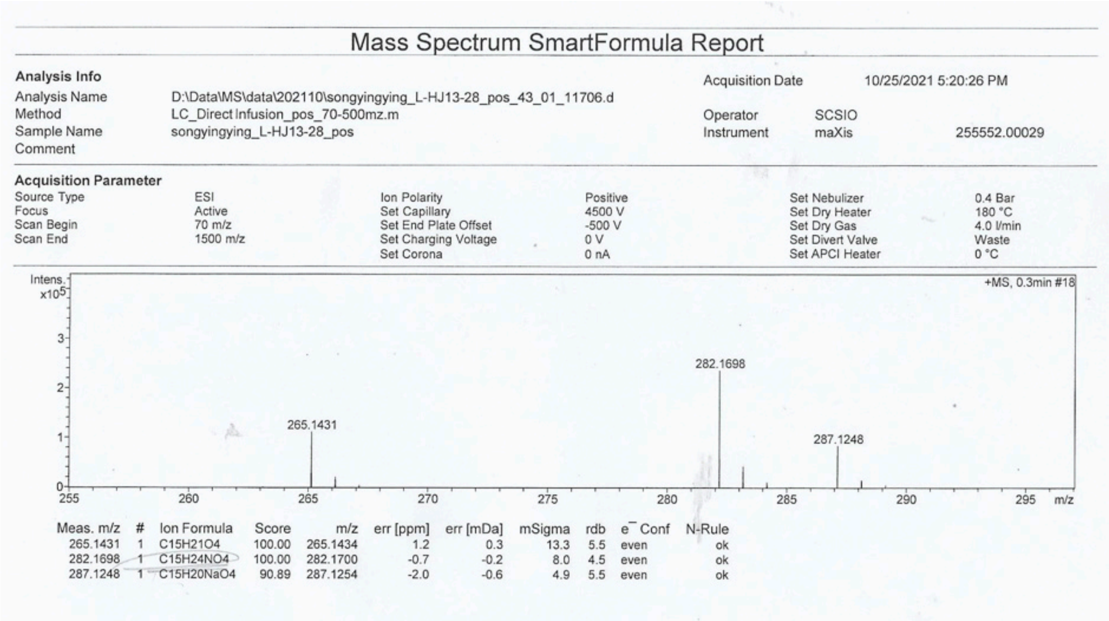

Mass Spectrum SmartFormula Report

Analysis Info

Analysis Name D:\Data\MS\data\202110\songyingying_L-HJ13-28_pos_43_01_11706.d
Method LC_Direct Infusion_pos_70-500mz.m
Sample Name songyingying_L-HJ13-28_pos
Comment

Acquisition Date 10/25/2021 5:20:26 PM
Operator SCSIO
Instrument maXis 255552.00029

Acquisition Parameter

Source Type	ESI	Ion Polarity	Positive	Set Nebulizer	0.4 Bar
Focus	Active	Set Capillary	4500 V	Set Dry Heater	180 °C
Scan Begin	70 m/z	Set End Plate Offset	-500 V	Set Dry Gas	4.0 l/min
Scan End	1500 m/z	Set Charging Voltage	0 V	Set Divert Valve	Waste
		Set Corona	0 nA	Set APCI Heater	0 °C

Meas. m/z	#	Ion Formula	Score	m/z	err [ppm]	err [mDa]	mSigma	rdb	e⁻ Conf	N-Rule
265.1431	1	C15H21O4	100.00	265.1434	1.2	0.3	13.3	5.5	even	ok
282.1698	1	C15H24NO4	100.00	282.1700	-0.7	-0.2	8.0	4.5	even	ok
287.1248	1	C15H20NaO4	90.89	287.1254	-2.0	-0.6	4.9	5.5	even	ok

Figure S46 HRESIMS spectrum of compound **5**

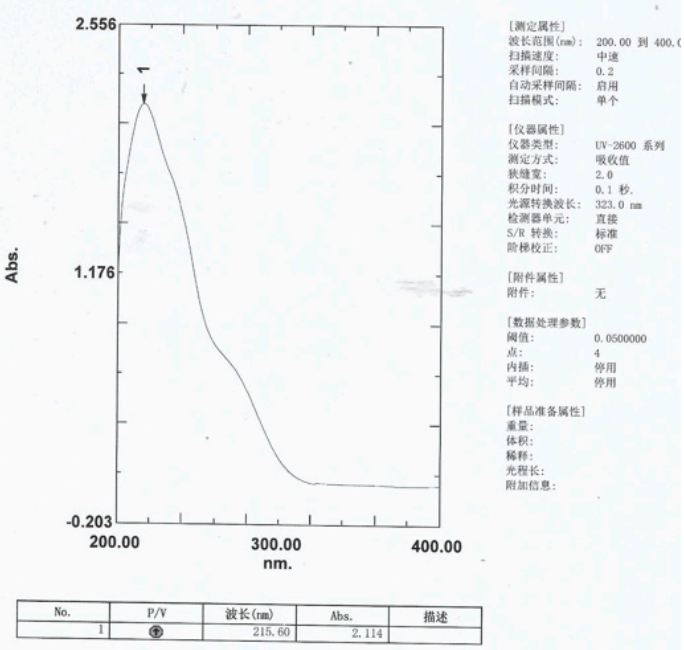

Figure S47 UV spectrum of **5**

Table S1 Energies of **1** and **2** at MMFF94 force field

Conformer	Energy (kcal/mol)	ΔE (kcal/mol)	Population (%)
13*R*, 14*R*, 15*R*-**1**	22.42	0.00	100.00
13*S*, 14*R*, 15*R*-**1**	30.13	0.00	100.00
13*R*, 14*R*, 15*R*-**2**	32.42	0.00	100.00

Table S2 Energies of **1** and **2** at B97-3c level in methanol

Conformer	Configuration	E (Hartree)	Energy (kcal/mol)	ΔE (kcal/mol)	Population (%)
13*R*, 14*R*, 15*R*-**1**	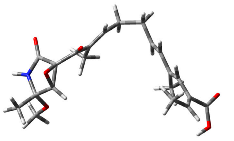	-2529	-1392032.79	0.00	100.00
13*S*, 14*R*, 15*R*-**1**	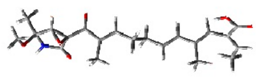	-2390	-1499748.9	0.00	100.00
13*R*, 14*R*, 15*R*-**2**	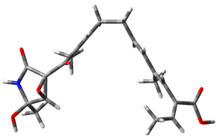	-2379	-1485709.29	0.00	100.00
